# Homogeneous Datasets of Triple Negative Breast Cancers Enable the Identification of Novel Prognostic and Predictive Signatures

**DOI:** 10.1371/journal.pone.0028403

**Published:** 2011-12-29

**Authors:** Thomas Karn, Lajos Pusztai, Uwe Holtrich, Takayuki Iwamoto, Christine Y. Shiang, Marcus Schmidt, Volkmar Müller, Christine Solbach, Regine Gaetje, Lars Hanker, Andre Ahr, Cornelia Liedtke, Eugen Ruckhäberle, Manfred Kaufmann, Achim Rody

**Affiliations:** 1 Department of Obstetrics and Gynecology, J. W. Goethe-University, Frankfurt, Germany; 2 Department of Breast Medical Oncology, University of Texas M.D. Anderson Cancer Center, Houston, Texas, United States of America; 3 Department of Obstetrics and Gynecology, Gutenberg-University, Mainz, Germany; 4 Department of Obstetrics and Gynecology, University Hospital Hamburg-Eppendorf, Hamburg, Germany; 5 Department of Obstetrics and Gynecology, University of Muenster, Muenster, Germany; 6 Department of Obstetrics and Gynecology, Saarland-University, Homburg, Germany; Macquarie University, Australia

## Abstract

**Background:**

Current prognostic gene signatures for breast cancer mainly reflect proliferation status and have limited value in triple-negative (TNBC) cancers. The identification of prognostic signatures from TNBC cohorts was limited in the past due to small sample sizes.

**Methodology/Principal Findings:**

We assembled all currently publically available TNBC gene expression datasets generated on Affymetrix gene chips. Inter-laboratory variation was minimized by filtering methods for both samples and genes. Supervised analysis was performed to identify prognostic signatures from 394 cases which were subsequently tested on an independent validation cohort (n = 261 cases).

**Conclusions/Significance:**

Using two distinct false discovery rate thresholds, 25% and <3.5%, a larger (n = 264 probesets) and a smaller (n = 26 probesets) prognostic gene sets were identified and used as prognostic predictors. Most of these genes were positively associated with poor prognosis and correlated to metagenes for inflammation and angiogenesis. No correlation to other previously published prognostic signatures (recurrence score, genomic grade index, 70-gene signature, wound response signature, 7-gene immune response module, stroma derived prognostic predictor, and a medullary like signature) was observed. In multivariate analyses in the validation cohort the two signatures showed hazard ratios of 4.03 (95% confidence interval [CI] 1.71–9.48; P = 0.001) and 4.08 (95% CI 1.79–9.28; P = 0.001), respectively. The 10-year event-free survival was 70% for the good risk and 20% for the high risk group. The 26-gene signatures had modest predictive value (AUC = 0.588) to predict response to neoadjuvant chemotherapy, however, the combination of a B-cell metagene with the prognostic signatures increased its response predictive value. We identified a 264-gene prognostic signature for TNBC which is unrelated to previously known prognostic signatures.

## Introduction

Breast cancer represents a heterogeneous disease and the currently most relevant clinical classification is based on the expression of the estrogen receptor (ER), progesteron receptor (PgR), as well as the human epidermal growth factor receptor 2 (HER2) [1], [2]. Molecular analyses of breast cancer have led to the introduction of molecular subtypes that largely recapitulate this clinical classification schema [3], [4] even when studies directly comparing those two approaches for individual samples have shown considerable discrepancies [5], [6]. To develop clinically more useful novel markers it will be necessary to study the known subtypes separately to avoid rediscovering genes that are highly co-expressed with ER, PgR, and HER2 [7]. The presently available prognostic gene signatures for breast cancer mainly reflect proliferation status and are most useful in ER-positive cancers [4]. For triple negative breast cancers (TNBC) [8] which lack the expression of all three receptors and represent an aggressive disease the use of these molecular prognostic signatures is limited.

In previous studies we demonstrated that analysis of a cohort of only TNBC allows the identification of different molecular phenotypes within this subtype of breast cancer [9], [10]. For the current study we assembled all publically available TNBC gene expression datasets generated on Affymetrix gene chips to achieve the largest possible size for prognostic marker discovery and validation. To minimize inter-laboratory variation only highly comparable arrays were included and dataset-biased genes were also removed. We partitioned the data into a discovery (i.e finding) and validation cohort and used a supervised approach to develop prognostic signatures. We also assessed the correlation between the resulting prognostic predictors with 16 previously described metagenes that can be used to categorize TNBC into molecular subsets [9], [10]. The prognostic signatures showed the highest correlation with the Interleukin-8(IL-8)/inflammation, Vascular endothelial growth factor (VEGF), and Histone metagenes. However, the signatures did not correlate with previously published prognostic signatures. The majority of prognostic genes that we identified were associated with poor prognosis, the few genes associated with good prognosis were mainly genes that correlated with immune cell metagenes.

## Materials and Methods

**Figure 1:**
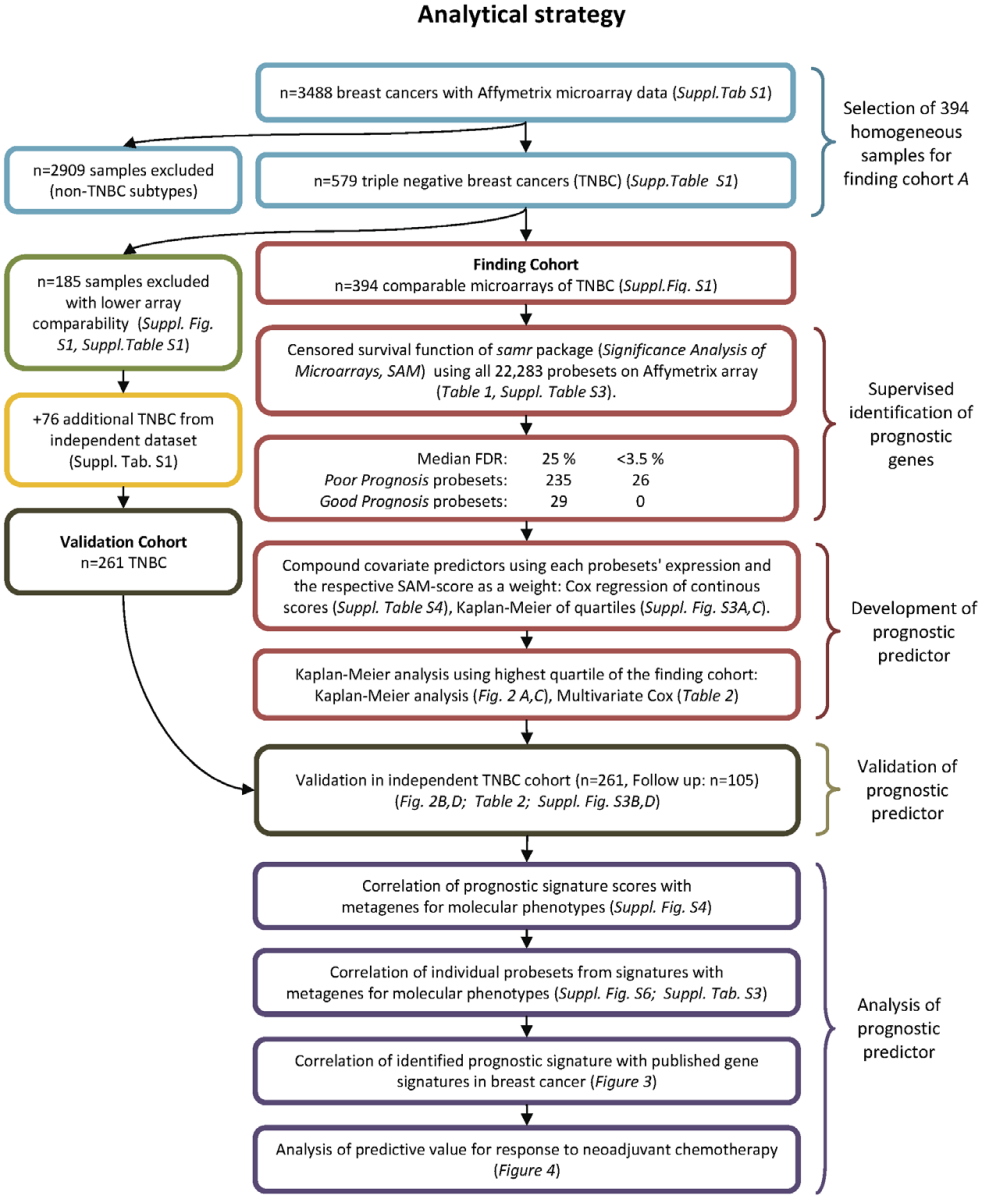
Development and validation of prognostic predictors according to REMARK criteria (McShane et al. J Clin Oncol. 2005;23:9067). The outline of the analysis strategy is schematically shown. The upper part shows the selection of the homogenous sample cohort of 394 TNBC. The middle part shows the identification of prognostic genes for TNBC, the development of the prognostic predictor, and the validation of this gene signature. The lower part displays the analysis of the genes which make up the signature regarding their relationship to previous known molecular factors among TNBC.

The REMARK recommendations for tumor marker studies [11] were applied in all analyses of this study. The analytical strategy and use of samples is illustrated in Figure 1, including the number of cases used in each stage of the analysis. The R software environment [12] (http://www.r-project.org/) and SPSS version 17.0 (SPSS Inc., Chicago, Illinois) were used for all analyses. Chi square test was applied to assess associations between categorical parameters. All reported P values are two sided and P≤0.05 was considered significant. An R script of the analyses is available as Data S1 with accompanying data in an R.Data file as Data S2.

### Assembly of a combined Affymetrix dataset from triple negative breast cancers

To generate a homogeneous dataset for the identification of genes with prognostic power among TNBC we used (i) only one array platform (Affymetrix U133 gene chips) and (ii) included only samples defined as triple negative based on the mRNA expression levels of ER, PgR, and HER2 as previously described [13], [14]. The assembly of the finding cohort of 394 TNBC samples has been reported previously [9], [10]. This yielded gene expression data from n = 3488 primary breast cancers including 28 different datasets (Table S1). The data was processed with the MAS5.0 algorithm [15] of the *affy* package [16] of the Bioconductor software project [17]. Data from each array were log_2_-transformed, median-centered, and the expression values of all the probesets from the U133A array were multiplied by a scale factor *S* so that the magnitude (sum of the squares of the values) equals one. Within this large breast cancer dataset, 579 triple negative breast cancers (TNBC) were identified based on the expression of ER, PgR, and HER2 from microarray [14]. The complete normalized expression data of the 579 TNBC is available from Gene Expression Omnibus as supplementary file, accession number GSE31519. In addition raw microarray data of all new samples and all relationships to re-analyzed samples are given under this accession. Next, we calculated a comparability metric *C* for each of the 579 arrays to identify the most comparable samples. This metric *C* is derived from the sum of the squared differences of the mean (*μ*) within a specific dataset and among all datasets, respectively, normalized by the standard deviation (σ) calculated for all genes (*g*) on the array:
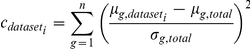



All datasets were sorted according to this metric and the top 15 datasets with the lowest values (norm. *C*≤0.03), corresponding to 394 samples in total, were used as the discovery cohort (Figure S1). The remaining 185 samples with lower array comparability together with an additional set of 76 TNBC samples that were obtained from an independent cohort of breast cancers [18] were used for validation (n = 261) (see Figure 1).

### Supervised prognostic signature generation by SAM

We applied a supervised classification method using all 22,283 probesets on the Affymetrix microarrays to identify a prognostic gene expression signature. The *Cox* score option of *Significance Analysis of Microarrays* (SAM) [19] using the R-package *samr* was applied to the finding cohort of 297 TNBC samples with known follow up to train the predictor. Delta values of 0.3 and 0.5 with median false discovery rates of 25% and <3.5%, respectively, were used to select prognostic probesets and a compound covariate predictor was developed that used the SAM-Score as a weight for each corresponding probeset. For Kaplan-Meier analysis we split the cases into quantiles of prediction scores and plotted survival curves by quartiles and also for the highest quartile versus all the rest.

### Assessment of dataset bias among probesets with prognostic value

Informative probesets obtained by SAM analysis were checked for dataset bias (i.e. differential expression by dataset of origin that would indicate laboratory-bias or sampling differences compared to the rest). To assess dataset bias, we used Kruskal Wallis statistic comparing the expression of each probeset with the primary dataset vector across the 394 TNBC. Each probeset was then tagged with that Kruskal Wallis value throughout all analyses (Figure S5). Cutoffs for exclusion of probesets due to strong dataset bias were derived from the distribution of the Kruskal Wallis statistic over all datasets for each probeset (Figure S2). Those cutoff values were used in stability analyes to validate the robustness of the obtained results (Figure S8).

### Correlation of prognostic genes with molecular phenotypes of TNBC

To determine if the genes (i.e probesets) from the prognostic signature correspond to or serve as surrogates for previously described molecular subtypes within the TNBC group, we calculated the correlation between each of the genes from the prognostic gene lists and 16 previously established metagenes that represent different cell populations and different molecular variants of TNBC. These metagenes included the intrinsic genes of the basal-molecular class [3], an apocrine/androgen receptor signalling signature [20], [21], five signatures related to different types of immune cells [22], [23], [24], [25], a stromal signature [26], the claudin-CD24 signature [27], [28], [29], markers of blood [30] and adipocytes [3], as well as an angiogenesis signature [31], [23] and an inflammatory signature [32], [33], [34]. The discovery of these metagenes was published previously [9], [10] and probeset IDs are isted in Table S2. Metagene values were calculated as mean expression of all probesets that define the metagene. Both the compound prognostic signature scores as well as the individual expression of each of the probesets from the SAM lists were correlated with the expression values of the 16 metagenes. Probesets that did not correlate to any of the metagenes at a pre-specified cutoff (see Results section) were designated as “*unclassified*”.

### Correlation of the identified prognostic signature scores with published gene signatures in breast cancer

The correlation of the newly identified prognostic signatures with seven previously published prognostic signatures was analyzed by calculating the Pearson correlation coefficient between signature scores in the finding cohort of TNBC. The following prognostic signatures were included in this analysis: Recurrence score [35], genomic grade index [36], 70-gene signature [37], wound response signature [38], 7-gene immune response module [39], stroma derived prognostic predictor [40], and a medullary like signature [18]. The *genefu* R-package [41], [42] was used to calculate the signature score as continuous variables and these were visualized through hierarchical clustering including the current TNBC-derived prognostic signatures and all other previously described prognostic signatures and the 16 metagenes.

### Survival analyses

Follow-up data was available for 297 of the 394 TNBC samples from the finding cohort, and for 105 of the 261 samples from the validation cohort (Table S1). All survival intervals were measured from the time of surgery to the survival endpoint that was available for that dataset. In 11 datasets (n = 241), the end point was relapse free survival (RFS) and in 6 other dataset (n = 161) it was distant metastasis free survival (DMFS). RFS includes local recurrences as events whereas DMFS does not. In order to plot Kaplan-Meier survival curves and perform survival analysis of the pooled data, we combined both types of endpoints into a single event free survival (EFS) endpoint that includes either RFS or DMFS whichever is available for the particular case. We have previously shown that the effect of using these different endpoints was rather small in the overall dataset [14]. All results from the pooled survival analyses were also verified by examining the effect of the different endpoints in stratified analyses. Follow-up data for those women in whom the survival end point was not reached were censored at the last follow-up or at 120 months. Subjects with missing values were excluded. We constructed Kaplan-Meier curves and used the log-rank test to determine the univariate significance of the variables. Cox regression analysis was applied to analyze the univariate hazard ratio of individual metagenes as continous variables. A Cox proportional-hazards model was used to simultaneously examine the effects of multiple covariates on survival. The effect of each individual variable was assessed with the use of the Wald test and described by the hazard ratio and 95% confidence intervals (95% CI).

### Predictive value of prognostic genes for response to neoadjuvant chemotherapy in TNBC

A cohort of TNBC treated with neoadjuvant chemotherapy was assembled for which gene expression data from pre-treatment biopsies were available. Samples from biopsies which were microdissected were excluded. For 191 samples from seven datasets information on pathological complete remission (pCR) was available (see Table S5). Receiver operator characteristics (ROC) analyses was applied to test the value of the TNBC-derived prognostic signatures as predictors of pathological complete response (pCR) to neoadjuvant chemotherapy. The predictive value of the newly identified signatures was also compared to that of a B-cell metagene as well as a combination of both markers. We have previously demonstrated a strong correlation of B-cell and T-cell metagenes in breast cancers [22]. This result is in line with the observation by our group and others that lymphocyte infiltration in breast cancer generally represents a mixture of both B- and T-cells [22]. Consequently both B- and T-cell metagenes carry nearly identical information and can both be used as a surrogate marker for infiltration of both types of lymphocytes with similar results. Superiority of one of these markers generally results from the specific dataset and/or cutoff point used [9], [22]. In the TNBC cohort used in this study the B-cell metagene outperformed the T-cell metagene as a continous factor [9].

## Results

### Identification of prognostic markers within the subgroup of triple negative breast cancer

**Figure 2:**
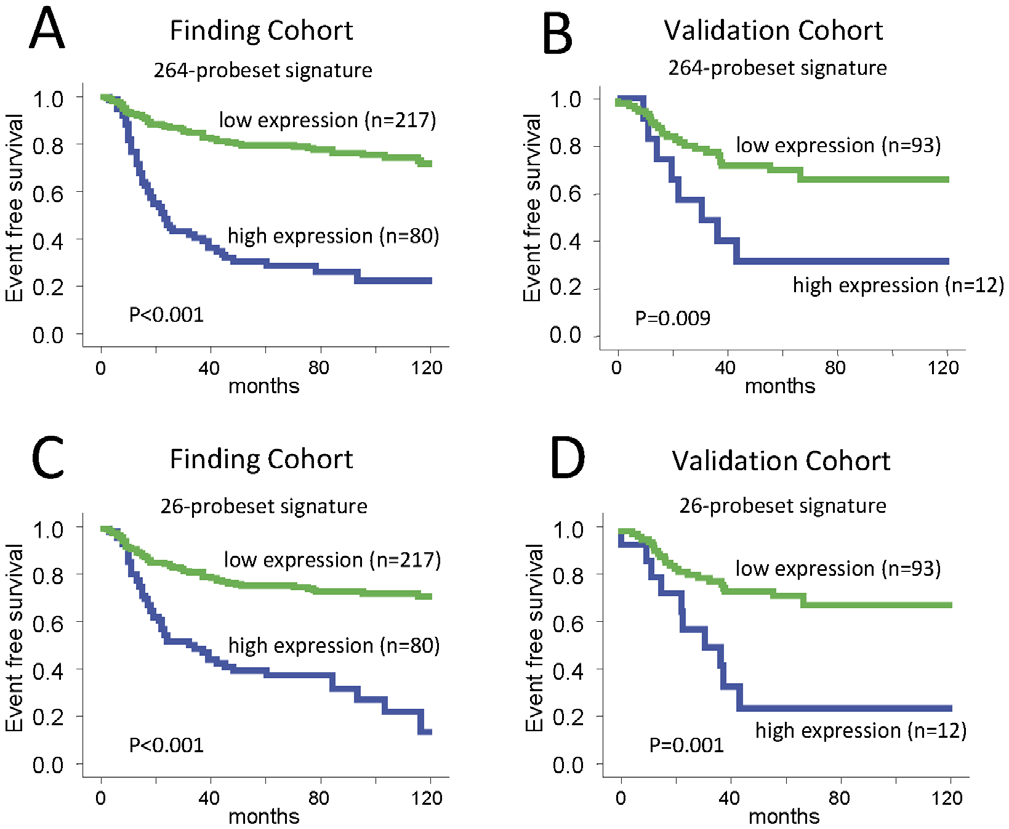
Kaplan Meier analysis according to the prognostic signatures in the finding and validation cohort. A) The 394 TNBC samples from the finding cohort were stratified according to the highest quartile of expression of the 264-probeset signature score. Kaplan Meier analysis of event free survival of 297 samples with follow up information is shown. B) The 261 TNBC samples from the validation cohort were stratified according to the highest quartile of expression of the 264-probeset signature score. Kaplan Meier analysis of event free survival of 105 samples with follow up information is shown. C) The same analysis as in (A) was performed using the 26-probeset signature. D) The same analysis as in (B) was performed using the 26-probeset signature.

The *Cox* score option of *Significance Analysis of Microarrays* (SAM) [19] of the R-package *samr* was applied to the finding cohort (n = 297 samples with follow up). A delta value of 0.3 resulted in 264 prognostic probesets (235 probesets associated with poor prognosis and 29 probesets associated with good prognosis). The median false discovery rate (FDR) when using this delta value was 25%. A more stringent delta of 0.5 resulted in 26 probesets associated with poor prognosis with a median false discovery rate <3.5% (Table 1). These 26 probesets are a subset of the larger 235 probesets list (Table S9). No probesets were associated with good prognosis at this higher stringency. The detailed results from the SAM analysis are given in Table S3. Two distinct signatures were constructed from the 264 and 26 probesets, respectively. The prognostic values of both signatures were highly significant in the finding cohort when analysed as a continous variable in multivariate Cox regression (Table S4). Inspection of the Kaplan Meier survival curves corresponding to the 4 prognostic score quartiles (for both the 264- and 26-gene predictors) suggested the highest quartile as a natural cutoff to dichotomize the patient population (Figure S3). This cutoff was used to plot survivals curves that are presented on Figure 2 and include the results for both the finding and the validation cohorts. Both signatures had strong and similar prognostic value in the discovery as well as in the validation datasets. Table 2 includes the corresponding multivariate Cox regression analyses of standard parameters and the prognostic signatures. In the validation cohort the stratification according to the 264-probeset signature resulted in a hazard ratio (HR) of 2.76 (95% CI 1.24–6.13; P = 0.013) in univariate analysis, and HR 4.03 (95% CI 1.71–9.48; P = 0.001) in multivariate analysis (Table 2). For the 26-probeset signature, in the validation cohort we observed a HR of 3.26 (95% CI 1.54–6.90; P = 0.002) in univariate, and HR 4.08 (95% CI 1.79–9.28; P = 0.001) in multivariate analysis. In the multivariate analyses only lymph node status (P = 0.048) retained its significance in the presence of the 26-probeset signature while age, tumor size, and histological grading did not reach significance (Table 2).

**Table 1:** 26 probeset supervised prognostic signature for TNBC from SAM.

Affy_ID	GeneSymbol	SAM-Score	direction of prognostic value (poor/good)
211506_s_at	IL8	3.754	POOR
211708_s_at	SCD	3.377	POOR
39249_at	AQP3	3.308	POOR
202859_x_at	IL8	3.299	POOR
202627_s_at	SERPINE1	3.136	POOR
212909_at	LYPDC1	3.118	POOR
200737_at	PGK1	3.090	POOR
204344_s_at	SEC23A	3.075	POOR
205810_s_at	WASL	3.071	POOR
217356_s_at	PGK1	3.031	POOR
215779_s_at	HIST1H2BG	3.017	POOR
212344_at	SULF1	3.008	POOR
209875_s_at	SPP1	3.002	POOR
219434_at	TREM1	2.982	POOR
219508_at	GCNT3	2.966	POOR
208881_x_at	IDI1	2.959	POOR
215427_s_at	ZCCHC14	2.958	POOR
214603_at	MAGEA2	2.956	POOR
219875_s_at	PNAS-4	2.951	POOR
204083_s_at	TPM2	2.948	POOR
218468_s_at	GREM1	2.937	POOR
204615_x_at	IDI1	2.902	POOR
212354_at	SULF1	2.858	POOR
218469_at	GREM1	2.836	POOR
212353_at	SULF1	2.809	POOR
202497_x_at	SLC2A3	2.797	POOR

**Table 2:** Multivariate Cox analyses of event free survival of TNBC according to standard parameters and expression of the 264-probeset signature and the 26-probeset signature.

		Finding Cohort				Validation Cohort			
Variable		No. of patients§	Hazard Ratio	95% CI	P-Value‡	No. of patients§	Hazard Ratio	95% CI	P-Value‡
**264-probeset signature**	*High* vs Low*	59 vs 178	4.44	2.82–6.99	**<0.001**	11 vs 85	4.03	1.71–9.48	**0.001**
Lymph node status	LNN vs LNP	210 vs 27	0.73	0.38–1.40	0.341	55 vs 41	0.50	0.23–1.09	0.080
Age	>50 vs ≤50	113 vs 124	0.73	0.47–1.15	0.176	60 vs 36	2.03	0.91–4.54	0.085
Tumor size	≤2 cm vs >2 cm	72 vs 165	1.00	0.60–1.64	0.964	21 vs 75	0.94	0.36–2.47	0.899
Histological grading	G3 vs G1&2	166 vs 71	1.13	0.69–1.87	0.622	71 vs 25	0.75	0.32–1.72	0.491
**26-probeset signature**	*High* vs Low*	62 vs 175	3.76	2.38–5.94	**<0.001**	15 vs 81	4.08	1.79–9.28	**0.001**
Lymph node status	LNN vs LNP	210 vs 27	0.71	0.37–1.36	0.300	55 vs 41	0.45	0.21–0.99	**0.048**
Age	>50 vs ≤50	113 vs 124	0.67	0.42–1.06	0.090	60 vs 36	1.87	0.84–4.16	0.125
Tumor size	≤2 cm vs >2 cm	72 vs 165	0.96	0.58–1.58	0.860	21 vs 75	0.97	0.37–2.53	0.946
Histological grading	G3 vs G1&2	166 vs 71	1.01	0.61–1.67	0.986	71 vs 25	0.68	0.29–1.59	0.372

§ information on all parameters was available for 237 of the 297 TNBC samples with follow up data from the finding cohort and 96 of the 105 TNBC samples with follow up data from the validation cohort.

‡ Significant P-Values are given in bold.

*highest quartile of expression score vs. rest (see Supplementary Table S4 for analysis of continous signature scores).

### Correlation of the prognostic signature scores with molecular phenotypes in triple negative breast cancer

Several investigators described molecular subgroups within TNBC defined by the variable expression of various metagenes (i.e. average expression of highly co-expressed genes). In order to examine if our TNBC-derived prognostic signatures correspond to previously described metagenes that were used to subdivide TNBC, we calculated the correlation between the our signature scores and 16 different previously published TNBC-related metagenes [9], [10]. Figure S4 displays the results of hierarchical clustering (based on Person correlation) of the 264-gene signature score and the different metagenes. The highest correlation was observed to the *VEGF*, *Histone*, and *IL-8* metagenes in the finding cohort (Figure S4 panel A). In the validation cohort, the *Stroma* and *Hemoglobin* metagenes also clustered within this group (Figure S4 panel B). Of note however, these latter two metagenes are associated with a high dataset bias (see Figure S5). A similar result was obtained with the 26-probeset signature which is shown in Figure S4 panel C and D. This signature also clustered together with *VEGF*, *IL-8*, and *Histone* metagenes.

### Correlation of individual markers from the prognostic signatures with triple negative breast cancer metagenes

In order to examine if the individual genes that constitute the TNBC-derived prognostic signatures correspond to the previously described gene clusters within TNBC or represent new potential markers, we also calculated the correlation between each individual probeset from the supervised signatures and the 16 TNBC-related metagenes. Figure S6 shows a heat map of the correlation matrix corresponding to the 264 probesets (235 associated with poor prognosis and 29 with good prognosis in panel A and B, respectively) and 16 metagenes in the 394 TNBC samples. The highest correlation coefficient for each of the probesets and the 16 metagenes is given in Table S3. A correlation coefficient ≥0.2 was used as threshold to assign a probeset to a specific metagene as correlated (Figure S6 panel A and B). Sixty eight of the 264 probesets (25.8%) showed correlation <0.2 to any metagene and these were designated as “*unclassified*” (Figure S6 panel A; alternatively we also applied a more stringent correlation coefficient cutoff ≥0.3 for a stability analysis which is shown in Figure S6 panel C and D). Of the 235 probesets that were associated with a poor prognosis, the largest probeset groups that were assigned to metagenes included *Stroma-*related (n = 51, 21.7%), *Histone*-related (n = 23, 9.8%), *Molecular-Apocrine*–related (n = 21, 8.9%), *Proliferation*–related (n = 17, 7.2%), and *IL-8/inflammation*–related (n = 13, 5.5%) (Table S3 and Figure S6 panel A). In contrast 21 of the 29 probesets (72.4%) associated with good prognosis were assigned to five metagenes each related to immune cell infiltration (*B-cell*, *T-cell*, *MHC-1*, *MHC-2*, and *IFN* metagenes; Figure S6 panel B).

### Correlation of the identified prognostic signature scores with published gene signatures in breast cancer

**Figure 3:**
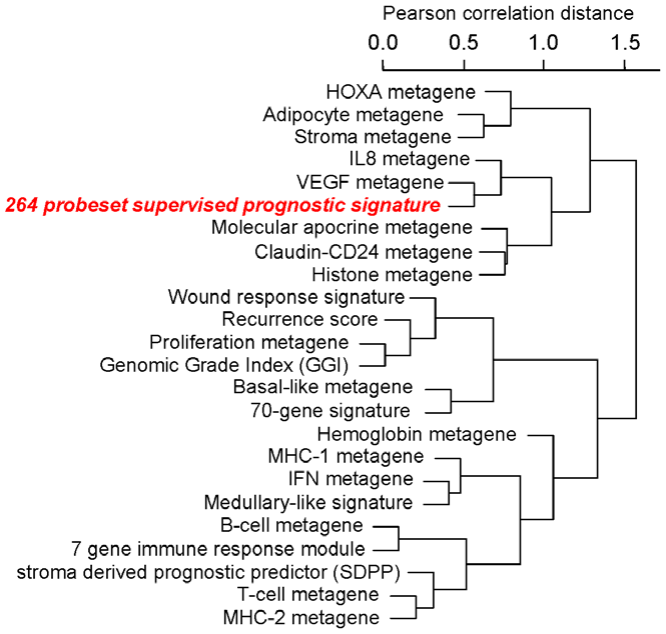
Relationship of the 264 probeset signature to the 16 metagenes and seven known prognostic signatures in TNBC. The 394 TNBC samples were analyzed for the expression of 16 metagenes and seven previously published prognostic signatures (recurrence score, genomic grade index, 70-gene signature, wound response signature, 7-gene immune response module, stroma derived prognostic predictor, and a medullary like signature). Resulting continous scores were used for hierarchical clustering using the Pearson correlation as a distance metric. The mutual relationships of all signatures is presented by the hierarchical dendrogram.

Several gene signatures were previously described that are predictive of prognosis of breast cancer in general. We also examined if our TNBC-derived signatures represent a surrogate of these previously reported breast cancer prognostic signatures including the recurrence score [35], the genomic grade index [36], the 70-gene signature [37], the wound response signature [38], the 7-gene immune response score [39], the stroma derived prognostic predictor [40], and a medullary like signature [18]. We assessed the correlation between our signatures and these signatures in our finding cohort. Figure 3 shows hierarchical clustering result of the 264-probeset signature score as continuous variable and the 16 metagenes and the seven previously published prognostic gene signatures. The recurrence score, the genomic grade index, the wound response signature, and the 70-gene signature, all clustered together with the proliferation and the basal-like metagenes. This indicates that many of the genes included in these signatures are related to proliferation. In contrast, the stroma derived prognostic predictor, the 7 gene immune response score, and the medullary-like signature clustered together with the different immune cell metagenes in a second large cluster. None of these signatures were related to our new TNBC-derived prognostic signature which clustered together with the *VEGF*-, *IL-8*-, *Molecular apocrine*-, *Claudin-CD24*-, and *Histone*-metagenes in a separate cluster (Figure 3). Similar results were obtained when we used the smaller 26-probeset signature (Figure S7).

### Predictive value of prognostic genes for response to neoadjuvant chemotherapy in TNBC

**Figure 4:**
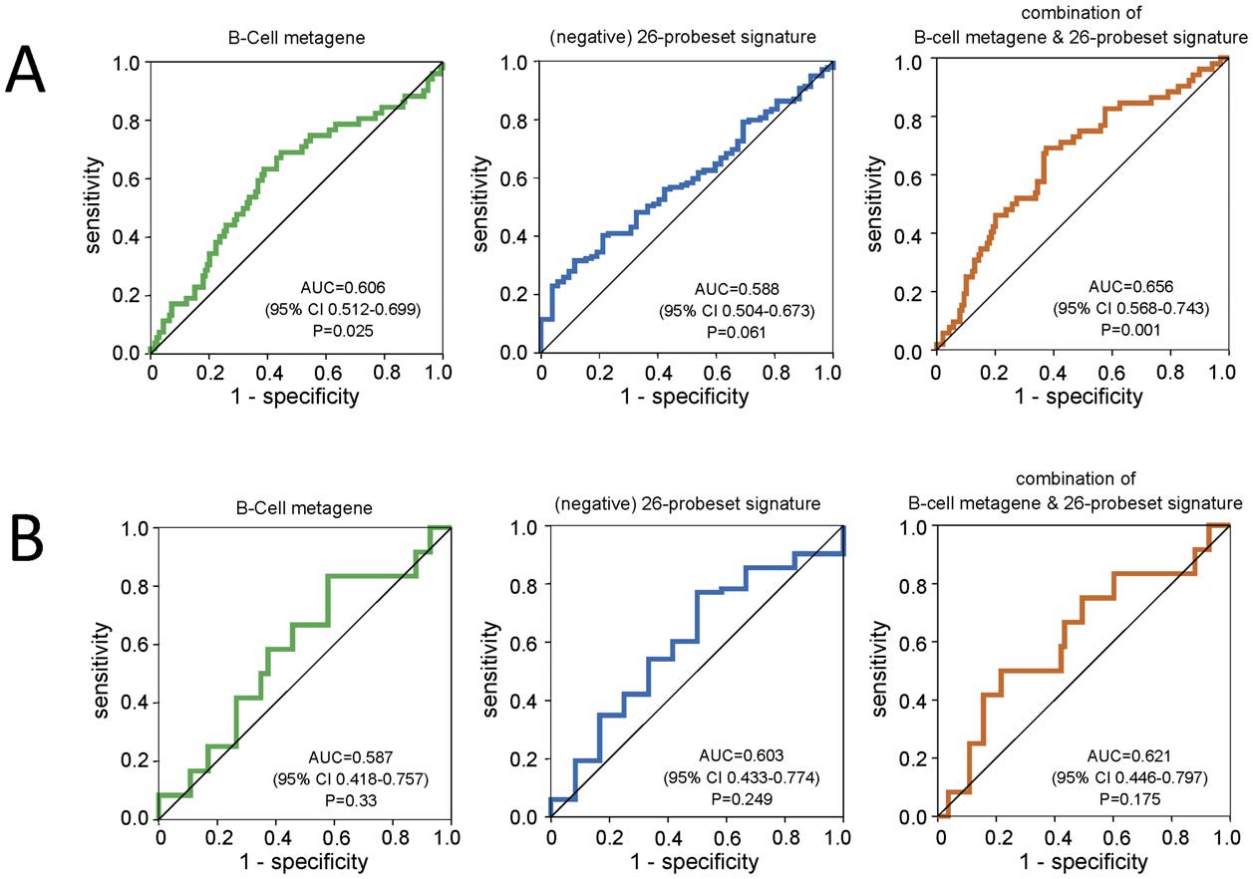
Analysis of the predictive value of an immune cell metagene and the supervised prognostic signature for response to neoadjuvant chemotherapy in TNBC. A) Neoadjuvant treated TNBC samples with information on pathological complete response (pCR) and available Affymetrix expression data were assembled from 7 datasets (MDA133, GSE16716, GSE18728, GSE19697, GSE20194, GSE20271, Frankfurt-3). Only pretherapeutic biopsies that were not microdissected were included (n = 191 nonredundant samples) of which 52 (27%) experienced a pCR. Three separate ROC curves for prediction of pCR by the B-Cell metagene, no-pCR by the 26-probeset signature, and pCR by a combination of both gene signatures are shown. The areas under the curve (AUC) were 0.606, 0.588, and 0.656, respectively. B) The same analyses as presented in (A) were performed using a smaller independent validation cohort of 95 TNBC from the TOP-Trial (GSE16446). AUC of 0.587, 0.603, and 0.621, respectively, and only a trend towards significance (P = 0.175) was observed in these data.

We have previously shown that tumor infiltration by lymphocytes reflected in the high expression of B-Cell and T-Cell metagenes in the cancer is predictive of response to neoadjuvant chemotherapy [22]. This predictive value was observed for both ER-positive and ER-negative cancers [22]. To test the potential predictive value of our newly identified prognostic signatures we assembled gene expression data from TNBC treated with neoadjuvant chemotherapy encompassing 191 samples that also had pathological complete response (pCR) data available (Table S5). Figure 4A shows the results of receiver operator characteristics (ROC) analyses for a previously published B-cell metagene which has known predictive value and for the 26-gene TNBC-derived prognostic signature. The area under the curve (AUC) for the B-cell metagene was 0.606 (95% CI 0.512–0.699, P = 0.025) and for the 264-gene signature it was 0.588 (95% CI 0. 504–0.673, P = 0.061). A simple linear combination of both scores led to an improved AUC of 0.656 (95% CI 0.568–0.743, P = 0.001). A similar but non-significant trend was seen in a separate 95 TNBC samples from the TOP-trial [43] (Table S5). In this independent validation dataset, the AUC of the B-cell metagene alone was 0.587 (95% CI 0.418–0.757, P = 0.33; Figure 4B) and it was 0.621 (95% CI 0.446–0.797, P = 0.175) for the combination of the 26-probeset signature and the B-cell metagene.

## Discussion

We identified two prognostic signatures including 264 and 26 probe sets each from gene expression data of triple negative breast cancers (TNBC) using a supervised discovery method. The smaller signature based on probe sets with the lowest false discovery rate represent a subset of the larger signature. We validated the independent prognostic value of both signatures in a separate validation cohort both using the signatures as continuous scores (P<0.0001; Table S4) as well as dichotomous variables (P = 0.001; Table 2). These gene signatures remained statistically significant prognostic predictors in multivariate analysis that included age, tumor size, nodal status and histologic grade. In order to develop these signatures we used TNBC cases only. Previous attempts to develop prognostic predictors almost invariable used mixed patient cohorts [37], [44], [45], [46], [47], [48], [49], [50]. The resulting signatures from those studies have frequently mirrored the differences in prognosis between molecular subtypes of breast cancer and were mainly associated with ER status and proliferation [4]. Consequently our new TNBC-derived prognostic signatures did not closely relate to the published general prognostic signatures (Figure 3). In contrast, the new signatures are mostly related to two metagenes which we previously described in TNBC, the IL-8/inflammation and VEGF metagenes. These metagenes were discovered through unsupervised analysis of the same dataset and are based on strong and consistent co-expression patterns and provided us with a tool to subclassify TNBC in a previous publication [9], [10]. Recent laboratory studies have demonstrated that IL-8 could directly increase the survival of breast cancer stem cells after chemotherapy [51] which can be blocked with IL-8 directed drugs [52]. The cytokine loops and cellular pathways regulated by IL-8 closely resemble those activated during chronic inflammation and wound healing which have previously been implicated in cancer [53].

A signature highly similar to our VEGF metagene was also described in an independent dataset recently [31]. In that study the VEGF metagene demonstrated high expression in metastatic breast cancer samples and was significantly associated with poor outcome in both breast and lung cancer and glioblastomas. These observations are consistent with our findings. Interestingly many of the genes included in VEGF metagene contain HIF1α binding sites and are known to be transcriptionally regulated by this hypoxia-induced factor and therefore may represent a molecular measure of tumor hypoxia [31]. This raises the possibility that the VEGF metagene and our prognostic signature that is related to it may only be a surrogate of increasing tumors size. But this seems not to be the case since we observed a negative correlation between the prognostic signature and tumors size (Table S6).

The 264-probest signature contains 29 probesets (11.0%) which were inversely associated with a poor prognosis and therefore we refer to it as good prognosis genes. Twenty one of these (72.4%) were correlated with immune cell metagenes which is consistent with several other publications which have shown that lymphocyte infiltration of TNBC is associated with an improved prognosis [22], [24], [25], [39], [18]. Metagenes which serve as surrogate markers for lymphocyte infiltration of the tumor (e.g. the B-Cell and T-Cell metagenes) are also predictive of response to neoadjuvant chemotherapy [22]. Therefore, we also assessed the chemotherapy predictive value of our prognostic signatures and found that it had only a week association with response to chemotherapy (Figure 4).

Our study has several limitations. The definition of TNBC was based on gene expression data which is not the standard definition used in the clinic. This definition holds the promise that samples erroneously characterized as receptor-negative by immunohistochemistry do not introduce noise into our analysis but discrepancies to cohorts defined by immunohistochemistry can occur. We found agreement of ER status between immunohistochemistry and gene expression data for 444 (84.4%) of 526 samples (86.8% and 81.3% in the finding and validation cohorts, respectively). For PgR status we found agreement for 407 (87.5%) of 465 samples (84.8% and 90.5% in finding and validation cohort), and for HER2 agreement for 347 (94.3%) of 368 samples (94.4% and 94.1% in finding and validation cohort). Agreement for the status of all three receptors was found for 276 (76.2%) of 362 samples (78.7% and 73.9% in finding and validation cohort, respectively). Regarding histological grading the proportion of grade 3 tumors is 73.5% and 74.1% in finding and validation cohort, respectively (Table S7). These numbers are clearly smaller than 92–98% in previously reported studies [8], [54], [55] indicating that the cohorts used in our study may not be truly representative of triple negative breast cancers in general. However despite the higher number of G1 and G2 samples histological grading was not a significant factor for survival in our cohort neither in multivariate nor univariate analysis. Most TNBC are high grade and therefore grade is not as important for prognosis in this subtype as it is in ER positive disease. Age and tumor size were also not significant in our cohorts, even in univariate analysis. TNBCs are also often associated with younger age but the impact of age for prognosis within this subtype is not yet fully clear. Several lines of evidence to suggest that tumour size may not be prognostic in TNBC [8], [56]. Still it cannot be excluded that a bias in our cohort is the reason for the lack of significance of these factors.

Our analysis involved pooling of several datasets to increase sample size and power for discovery and validation. This strategy bears the risk of a confounding effect through systematic technical differences that exist between individual datasets [57], [58]. To minimize this confounder we performed multiple filtering steps to remove biased datasets and dataset-biased genes (see Methods). In order to validate the robustness of the obtained results we also performed a stability analysis by using different filtering cutoffs (Figure S2). As shown in Figure S8 the validation of several alternative signatures generated through a variety of filtering combinations resulted in similar results in the validation cohort indicating a robust finding. This study also has the limitation of heterogeneous therapy of the cases included, some cancers were treated with surgery alone others received adjuvant or neoadjuvant chemotherapy of various types. This treatment heterogeneity limits the clinical interpretation of the findings, however since the prognostic signatures had limited predictive value for neoadjuvant chemotherapy response, we infer that their outcome predictive value is mostly derived from its prognostic components. However the “good” prognostic group still has more than 20% recurrence at 5 years. Thus this outcome would not change the actual clinical management of this subset of patients but could help to develop a clinically useful multivariate prognostic model for TNBC.

During the generation of this report Lehmann et al. [59] described a similar strategy of a pooled dataset of TNBC samples with microarray data. These authors identified seven different TNBC subtypes by unsupervised k-means clustering. The expression profiles of these subtypes are similar to many of the metagenes that we have reported for TNBC [9], [10]. Thus we wondered whether our supervised signature would also correlate with any of these subtypes. However as shown in Figure S9 no clear correlation of the supervised signature with any of these seven subtypes described by Lehmann et al. was observed. We have also analyzed whether our signature captures similar information as the well known intrinsic molecular subtypes of breast cancer [3], [60]. To this end we used a recently published implementation of different variants of the centroid method to assign single samples to a molecular subtype [61]. The corresponding results are shown in Table S8. We applied two alternative variants of the method which both led to the conclusion that no significant difference in subtype assignment was observed when samples were classified according to the expression of the 264-probeset signature.

In our previous study [9] we had used unsupervised methods to identify subgroups of TNBC without considering outcome in the first place. Based on subsequent correlation of the obtained groups with prognosis we then constructed a simple binary classifier from expression of B-cell- and IL-8-metagenes. In contrast, the supervised signature presented here seem to include information from several additional biological characteristics. In fact this supervised signature can outperform the simple combination of the two parameters used in our previous study. However, the interpretation of the biology of such an amalgamated signature could be much more difficult than the interpretation of metagenes.

In summary, in this paper we demonstrated that the use of a homogenous TNBC dataset allowed us to identify prognostic gene signatures that are unrelated to previously published general breast cancer prognostic signatures. The composition of the signature suggests that IL-8 mediated inflammation and VEGF related signaling herald very poor prognosis in TNBC and immune infiltration predicts better outcome. These observations could also suggest potential novel therapeutic strategies for these patients as e.g. inhibiting IL-8 signalling [51], [52] might be combined with anti-angiogenesis therapies [31], and immune augmentation [10].

## Supporting Information

Figure S1 **Selection of the TNBC finding cohort from multiple datasets based on dataset comparibility.** Triple negative breast cancers (TNBC, n = 579) from 28 datasets were sorted by dataset according to a dataset comparability metric (horizontally). Shown are the full array data of normalized Affymetrix U133A microarrays. The 15 most comparable datasets encompassing n = 394 TNBC samples were subsequently used as a finding cohort and the remaining 13 datasets (n = 185 TNBC samples) withhold as validation cohort.
(PDF)

Figure S2 **Analysis of a potential dataset bias among probesets of the prognostic signatures from the supervised analysis.** A) The standard Kruskal-Wallis rank test was used to analyze the dependence of each individual probesets' expression on the vector of the 15 different datasets in the finding cohort of n = 394 samples. The distribution of the rank sum statistics for all 22,283 probesets from the U133A array is shown. Two dotted vertical lines mark the used cutoff values of 75 (yellow) and 150 (red). B) Distribution of the Kruskal-Wallis rank sum statistics for the 235 probesets identified by SAM as associated with poor prognosis. Used cutoffs are represented by dotted vertical lines as in (A). C) Distribution of the Kruskal-Wallis rank sum statistics for th 29 probesets identified by SAM as associated with good prognosis. Used cutoffs are represented by dotted vertical lines as in (A).
(PDF)

Figure S3 **Kaplan Meier analysis of quartiles according to the prognostic signature scores in the finding and validation cohorts.** A) The 394 TNBC samples from the finding cohort were stratified according to quartiles of expression of the 264-probeset signature score. Kaplan Meier analysis of event free survival of 297 samples with follow up information is shown. B) The 261 TNBC samples from the validation cohort were stratified according to quartiles of expression of the 264-probeset signature score. Kaplan Meier analysis of event free survival of 105 samples with follow up information is shown. C) The same analysis as in (A) was performed using the 26-probeset signature. D) The same analysis as in (B) was performed using the 26-probeset signature.
(PDF)

Figure S4 **Correlation of the prognostic signatures with metagenes for molecular phenotypes in triple negative breast cancer.** A) The continous score of the 264-probeset signature was correlated with the expression of 16 metagenes for molecular phenotypes in the 394 TNBC samples from the finding cohort. Shown is the result from hierarchical average linkage clustering based on absolute Pearson correlation. The signature score clustered together with VEGF, Histone, and IL-8 metagenes. B) The same analysis as in (A) was performed in the validation cohort of 261 independent TNBC samples. In this analysis the signature score clustered together with Stroma, Hemoglobin, VEGF, and IL-8 metagenes. Of note, however, Stroma and Hemoglobin metagenes are associated with a high dataset bias (see Supplementary Figure S5). C) The same analysis as in (A) was performed with the 26-probeset signature in the 394 TNBC samples from the finding cohort. The 26-probeset signature which was obtained by higher stringency in SAM analysis clustered together with IL-8, VEGF, and Histone metagenes. D) The same analysis as in (C) was performed with the 26-probest signature in the validation cohort of 261 samples. Similar as in (C) the 26-probeset signature clustered together with VEGF, IL-8, Proliferation, and Histone metagenes.
(PDF)

Figure S5 **Analysis of dataset bias of metagenes and the prognostic signatures.** A) The dependence of earch probeset from the U133A array on the dataset vector was analyzed using the standard Kruskal-Wallis rank test in the finding cohort of 394 samples (see Suppl. Fig. S2). Box plots are shown for the Kruskal-Wallis statistics of the probesets of each metagene on the left and for the two prognostic signatures on the right. The highest dataset bias was observed for Stroma and Hemoglobin metagenes which is related to different applied biopsy methods (fine needle biopsy vs. surgical resection). B) The 261 samples from the validation cohort were used to calculate the Kruskal-Wallis rank sum statistics for all probesets. Again box plots are shown as in (A), but the Kruskal-Wallis statistics from the validation cohort were applied. Several metagenes are characterized by higher bias in the validation cohort.
(PDF)

Figure S6 **Correlation of individual markers from the prognostic signatures with known metagenes in triple negative breast cancer.** From the 264 Affymetrix probsets of the supervised prognostic signature, 235 probesets were associated with poor prognosis (analyzed in panels A and C) and 29 with good prognosis (analyzed in panels B and D). A) The 235 individual probesets associated with poor prognosis (horizontically) were analyzed for their correlation with the expression of 16 metagenes (vertically) for molecular phenotypes in the 394 TNBC samples from the finding cohort. 116 probesets displaying a Pearson correlation above a cutoff 0.2 are sorted (horizontically) on the left according to the assigned metagene while 60 probesets remained unclassified. B) The 29 individual probesets associated with good prognosis were analyzed as in (*A*) and 21 assigned to metagenes (cutoff 0.2) are sorted horizontically on the left while 8 remained unclassified. C) The same analysis as in (*A*) was performed using the more stringent cutoff 0.3 for assignment to a metagene resulting in 118 probesets correlated to metagenes from the list of 235 probesets associated with poor prognosis. D) The same analysis as in (*B*) was perfomed using the more stringent cutoff 0.3 resulting in 18 of the 29 good prognosis probesets assigned to metagenes. All individual correlation values are given in Supplementary Table S3.
(PDF)

Figure S7 **Relationship of the 26 probeset signature to the 16 metagenes and seven known prognostic signatures in TNBC.** The 394 TNBC samples were analyzed for the expression of 16 metagenes and seven previously published prognostic signatures as described in Figure 3 and hierachical clustered using Pearson correlation as distance metric. Abbreviations: SAMmean = 26 probeset signature wound.score$score = Wound response signature rs.394$score = recurrence score ggi.score$score = genomic grade index gene70.score$score = 70-gene signature sabatier.score$score = medullary like signature Tesch7.score$score = 7-gene immune response module sdpp.sore$score = stroma derived prognostic predictor.
(PDF)

Figure S8 **Stability analysis of the prognostic signatures from the supervised analysis.** The 264 Affymetrix probsets of the supervised prognostic signature were filtered according to their dataset bias measured through Kruskal-Wallis statistic and different stringency from SAM analysis as given in the Table below the graphs. The resulting probeset lists of 252, 24, 181, and 16 probesets were used for prognostic signature generation as the original 264 probeset list. In upper panels A, B, C, and D the correlation of the four alternative signatures to the 264-probeset signature is shown by scatter plot analysis. The lower panels display the results from the Kaplan-Meier analyses of the validation cohort of 261 TNBC (105 samples with follow up information). In addition P-Values of multivariate Cox regression analysis of the validation cohort using continous signature scores are given in the table below.
(PDF)

Figure S9 **Expression of the 264-probeset and 26-probeset signature scores in seven different TNBC subtypes according to Lehmann et al.** A) Box plots comparing the expression of the 264-probeset signature in the seven different TNBC subtypes according to Lehmann et al. (J Clin Invest. 2011; 121: 2750) separately for our finding and validation cohorts. No clear correlation of the expression of the signature with any of the subtypes was observed. The seven subtypes have been ordered according the expression of the signature in the finding cohort. Highest expression was observed in the “basal-like 2” (BL2) and “luminal androgen receptor” (LAR) subtypes of the finding cohort. However this effect was not reproduced in the validation cohort. B) The same analysis as in (*A*) was performed for the expression of the 26-probeset signature. The observed result was similar in that no reproducible correlation of the signature with any subtypes was detected.
(PDF)

Table S1 **Summary of Affymetrix microarray datasets used in this study.**
(PDF)

Table S2 **List of 355 Affymetrix probesets used for metagene calculation.**
(PDF)

Table S3 **Details of probesets from the supervised signatures.**
(XLS)

Table S4 **A) Multivariate Cox regression of continous 264-probeset signature and standard parameters for event free survival in the finding cohort B) Multivariate Cox regression of continous 26-probeset signature and standard parameters for event free survival in the finding cohort.**
(PDF)

Table S5 **Pre-therapeutic samples from neoadjuvant treated TNBC.**
(PDF)

Table S6 **Clinical parameters of TNBC according to expression of the 264-probeset signature.**
(PDF)

Table S7 **Histological grade among samples in the finding and validation cohort.**
(PDF)

Table S8 **Distribution of intrinsic molecular subtypes according to expression of the 264-probeset signature in TNBC.**
(PDF)

Table S9 **264 probeset supervised prognostic signature for TNBC from SAM.**
(PDF)

Data S1 **R script of the analyses.**
(R) Click here for additional data file.

Data S2 **R.Data file** (contains 11 data objects used in the R script from Data S1).
(7z) Click here for additional data file.
