# Supplementary material for: Homogeneous Datasets of Triple Negative Breast Cancers Enable the Identification of Novel Prognostic and Predictive Signatures
Source: PLoS One. 2011 Dec 29;6(12):e28403. doi: 10.1371/journal.pone.0028403 (PMC3248403; doi:10.1371/journal.pone.0028403)
Supplement: Figure S1 — Selection of the TNBC finding cohort from multiple datasets based on dataset comparibility. Triple negative breast cancers (TNBC, n = 579) from 28 datasets were sorted by dataset according to a dataset comparability metric (horizontally). Shown are the full array data of normalized Affymetrix U133A microarrays. The 15 most comparable datasets encompassing n = 394 TNBC samples were subsequently used as a finding cohort and the remaining 13 datasets (n = 185 TNBC samples) withhold as validation cohort. (PDF) [file pone.0028403.s001.pdf]

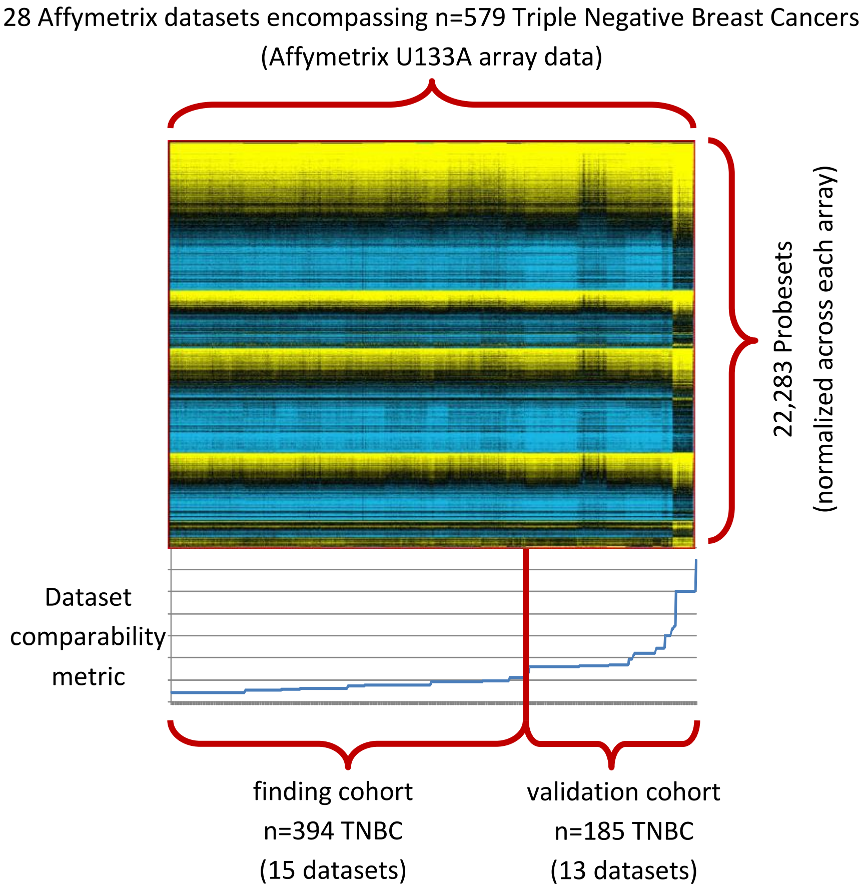

Supplementary Figure S1: Selection of the TNBC finding cohort from multiple datasets based on dataset comparibility

Triple negative breast cancers (TNBC, n=579) from 28 datasets were sorted by dataset according to a dataset comparability metric (horizontally). Shown are the full array data of normalized Affymetrix U133A microarrays. The 15 most comparable datasets encompassing n=394 TNBC samples were subsequently used as a finding cohort and the remaining 13 datasets (n=185 TNBC samples) withhold as validation cohort.
