# Supplementary material for: Homogeneous Datasets of Triple Negative Breast Cancers Enable the Identification of Novel Prognostic and Predictive Signatures
Source: PLoS One. 2011 Dec 29;6(12):e28403. doi: 10.1371/journal.pone.0028403 (PMC3248403; doi:10.1371/journal.pone.0028403)
Supplement: Figure S2 — Analysis of a potential dataset bias among probesets of the prognostic signatures from the supervised analysis. A) The standard Kruskal-Wallis rank test was used to analyze the dependence of each individual probesets' expression on the vector of the 15 different datasets in the finding cohort of n = 394 samples. The distribution of the rank sum statistics for all 22,283 probesets from the U133A array is shown. Two dotted vertical lines mark the used cutoff values of 75 (yellow) and 150 (red). B) Distribution of the Kruskal-Wallis rank sum statistics for the 235 probesets identified by SAM as associated with poor prognosis. Used cutoffs are represented by dotted vertical lines as in (A). C) Distribution of the Kruskal-Wallis rank sum statistics for th 29 probesets identified by SAM as associated with good prognosis. Used cutoffs are represented by dotted vertical lines as in (A). (PDF) [file pone.0028403.s002.pdf]

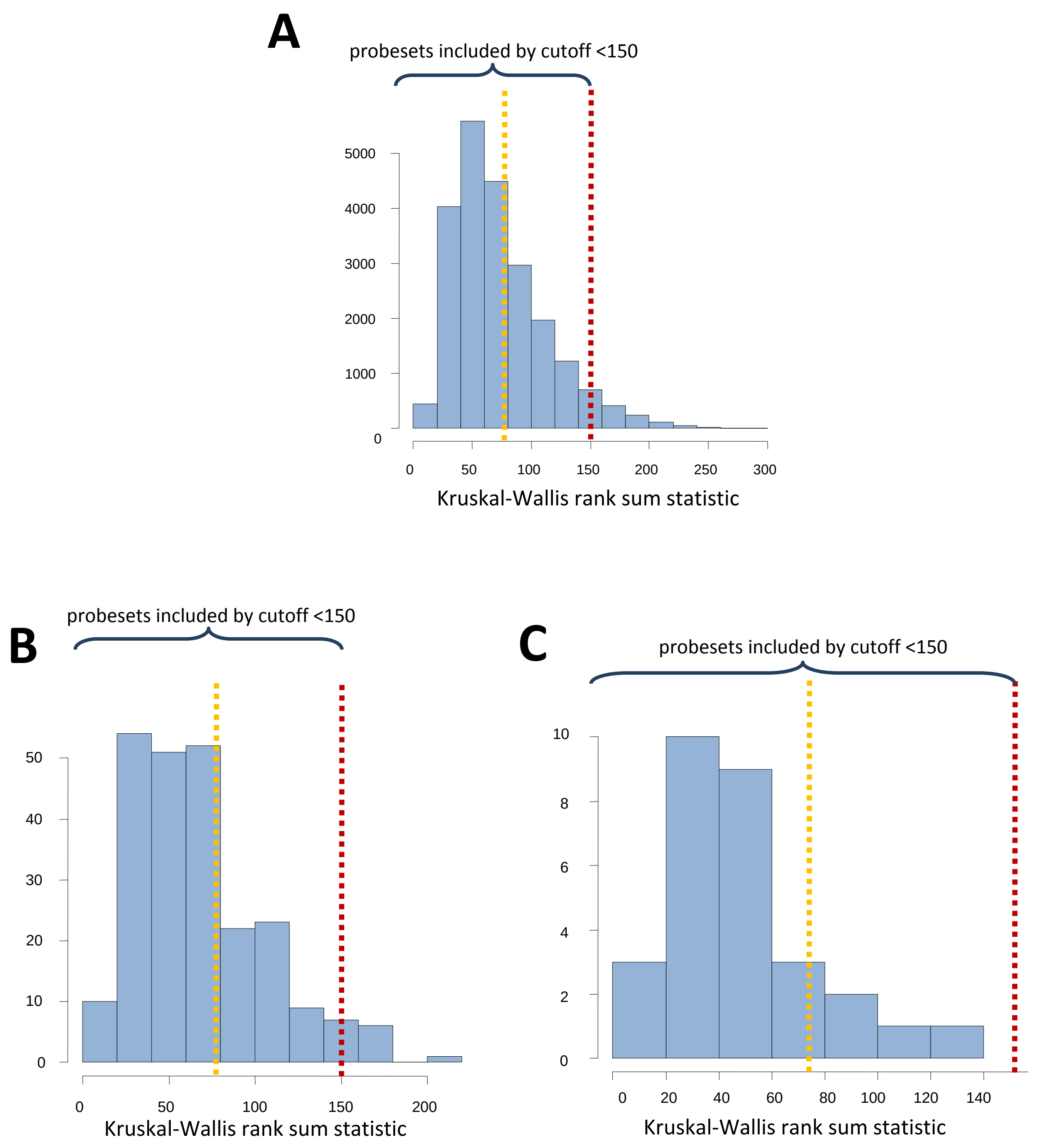

Supplementary Figure S2: Analysis of a potential dataset bias among probesets of the prognostic signatures from the supervised analysis

A) The standard Kruskal-Wallis rank test was used to analyze the dependence of each individual probesets' expression on the vector of the 15 different datasets in the finding cohort of n=394 samples. The distribution of the rank sum statistics for all 22,283 probesets from the U133A array is shown. Two dotted vertical lines mark the used cutoff values of 75 (yellow) and 150 (red).

B) Distribution of the Kruskal-Wallis rank sum statistics for the 235 probesets identified by SAM as associated with poor prognosis. Used cutoffs are represented by dotted vertical lines as in (A).

C) Distribution of the Kruskal-Wallis rank sum statistics for the 29 probesets identified by SAM as associated with good prognosis. Used cutoffs are represented by dotted vertical lines as in (A).
