# Supplementary material for: Homogeneous Datasets of Triple Negative Breast Cancers Enable the Identification of Novel Prognostic and Predictive Signatures
Source: PLoS One. 2011 Dec 29;6(12):e28403. doi: 10.1371/journal.pone.0028403 (PMC3248403; doi:10.1371/journal.pone.0028403)
Supplement: Figure S3 — Kaplan Meier analysis of quartiles according to the prognostic signature scores in the finding and validation cohorts. A) The 394 TNBC samples from the finding cohort were stratified according to quartiles of expression of the 264-probeset signature score. Kaplan Meier analysis of event free survival of 297 samples with follow up information is shown. B) The 261 TNBC samples from the validation cohort were stratified according to quartiles of expression of the 264-probeset signature score. Kaplan Meier analysis of event free survival of 105 samples with follow up information is shown. C) The same analysis as in (A) was performed using the 26-probeset signature. D) The same analysis as in (B) was performed using the 26-probeset signature. (PDF) [file pone.0028403.s003.pdf]

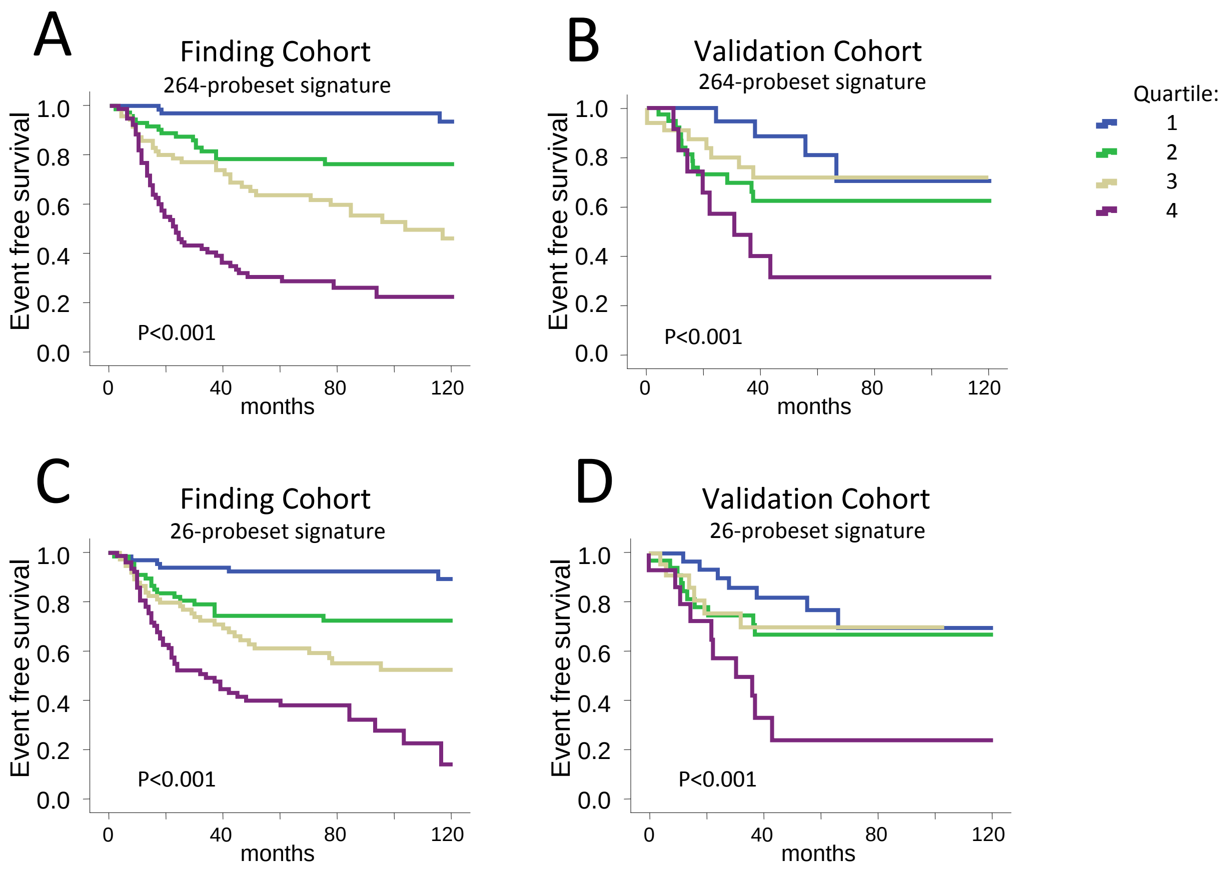

Supplementary Figure S3: **Kaplan Meier analysis of quartiles according to the prognostic signature scores in the finding and validation cohorts**

A) The 394 TNBC samples from the finding cohort were stratified according to quartiles of expression of the 264-probeset signature score. Kaplan Meier analysis of event free survival of 297 samples with follow up information is shown.
B) The 261 TNBC samples from the validation cohort were stratified according to quartiles of expression of the 264-probeset signature score. Kaplan Meier analysis of event free survival of 105 samples with follow up information is shown.
C) The same analysis as in (A) was performed using the 26-probeset signature.
D) The same analysis as in (B) was performed using the 26-probeset signature.
