# Supplementary material for: Homogeneous Datasets of Triple Negative Breast Cancers Enable the Identification of Novel Prognostic and Predictive Signatures
Source: PLoS One. 2011 Dec 29;6(12):e28403. doi: 10.1371/journal.pone.0028403 (PMC3248403; doi:10.1371/journal.pone.0028403)
Supplement: Figure S4 — Correlation of the prognostic signatures with metagenes for molecular phenotypes in triple negative breast cancer. A) The continous score of the 264-probeset signature was correlated with the expression of 16 metagenes for molecular phenotypes in the 394 TNBC samples from the finding cohort. Shown is the result from hierarchical average linkage clustering based on absolute Pearson correlation. The signature score clustered together with VEGF, Histone, and IL-8 metagenes. B) The same analysis as in (A) was performed in the validation cohort of 261 independent TNBC samples. In this analysis the signature score clustered together with Stroma, Hemoglobin, VEGF, and IL-8 metagenes. Of note, however, Stroma and Hemoglobin metagenes are associated with a high dataset bias (see Supplementary Figure S5). C) The same analysis as in (A) was performed with the 26-probeset signature in the 394 TNBC samples from the finding cohort. The 26-probeset signature which was obtained by higher stringency in SAM analysis clustered together with IL-8, VEGF, and Histone metagenes. D) The same analysis as in (C) was performed with the 26-probest signature in the validation cohort of 261 samples. Similar as in (C) the 26-probeset signature clustered together with VEGF, IL-8, Proliferation, and Histone metagenes. (PDF) [file pone.0028403.s004.pdf]

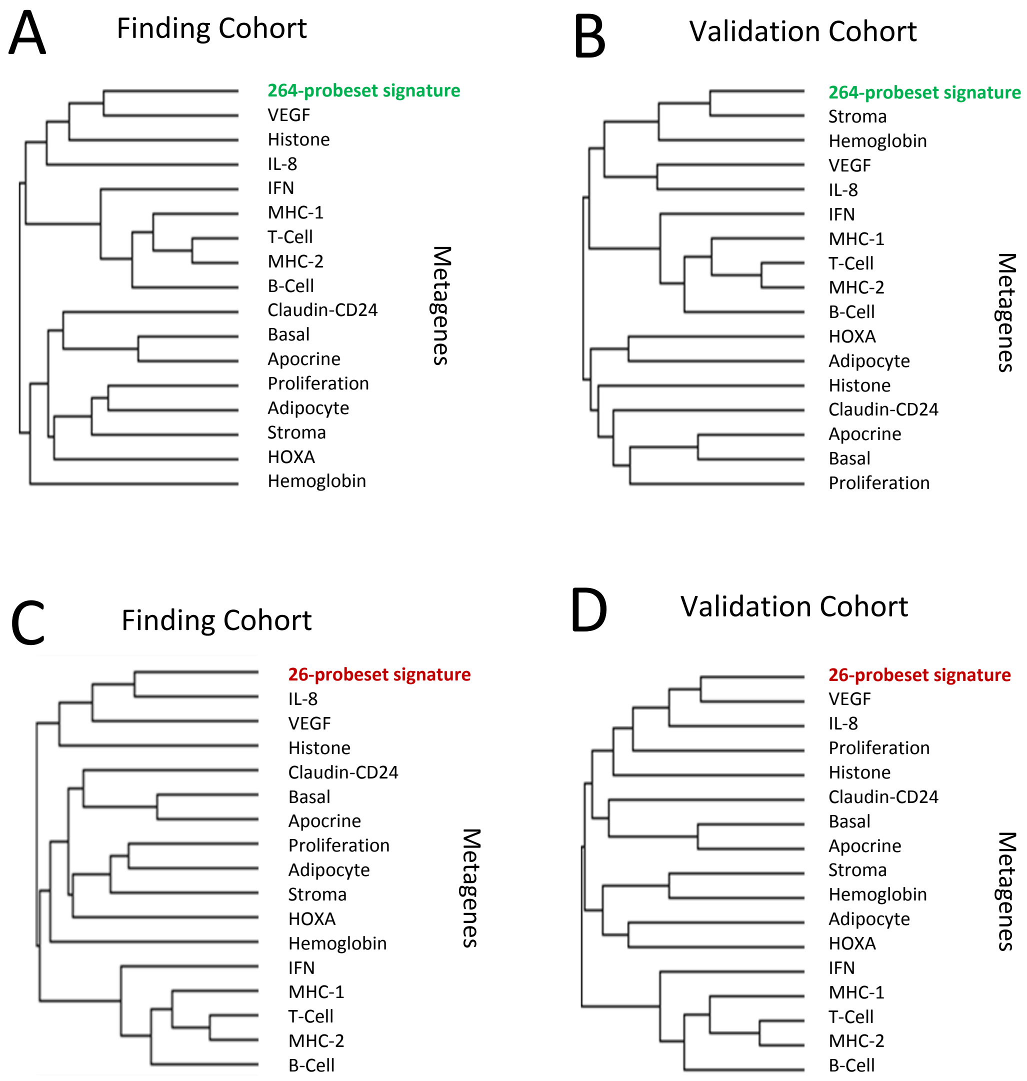

Supplementary Figure S4: Correlation of the prognostic signatures with metagenes for molecular phenotypes in triple negative breast cancer.

A) The continous score of the 264-probeset signature was correlated with the expression of 16 metagenes for molecular phenotypes in the 394 TNBC samples from the finding cohort. Shown is the result from hierarchical average linkage clustering based on absolute Pearson correlation. The signature score clustered together with VEGF, Histone, and IL-8 metagenes.

B) The same analysis as in (A) was performed in the validation cohort of 261 independent TNBC samples. In this analysis the signature score clustered together with Stroma, Hemoglobin, VEGF, and IL-8 metagenes. Of note, however, Stroma and Hemoglobin metagenes are associated with a high dataset bias (see Supplementary Figure S5).

C) The same analysis as in (A) was performed with the 26-probeset signature in the 394 TNBC samples from the finding cohort. The 26-probeset signature which was obtained by higher stringency in SAM analysis clustered together with IL-8, VEGF, and Histone metagenes.

D) The same analysis as in (C) was performed with the 26-probest signature in the validation cohort of 261 samples. Similar as in (C) the 26-probeset signature clustered together with VEGF, IL-8, Proliferation, and Histone metagenes.
