# Supplementary material for: Homogeneous Datasets of Triple Negative Breast Cancers Enable the Identification of Novel Prognostic and Predictive Signatures
Source: PLoS One. 2011 Dec 29;6(12):e28403. doi: 10.1371/journal.pone.0028403 (PMC3248403; doi:10.1371/journal.pone.0028403)
Supplement: Figure S5 — Analysis of dataset bias of metagenes and the prognostic signatures. A) The dependence of earch probeset from the U133A array on the dataset vector was analyzed using the standard Kruskal-Wallis rank test in the finding cohort of 394 samples (see Suppl. Fig. S2). Box plots are shown for the Kruskal-Wallis statistics of the probesets of each metagene on the left and for the two prognostic signatures on the right. The highest dataset bias was observed for Stroma and Hemoglobin metagenes which is related to different applied biopsy methods (fine needle biopsy vs. surgical resection). B) The 261 samples from the validation cohort were used to calculate the Kruskal-Wallis rank sum statistics for all probesets. Again box plots are shown as in (A), but the Kruskal-Wallis statistics from the validation cohort were applied. Several metagenes are characterized by higher bias in the validation cohort. (PDF) [file pone.0028403.s005.pdf]

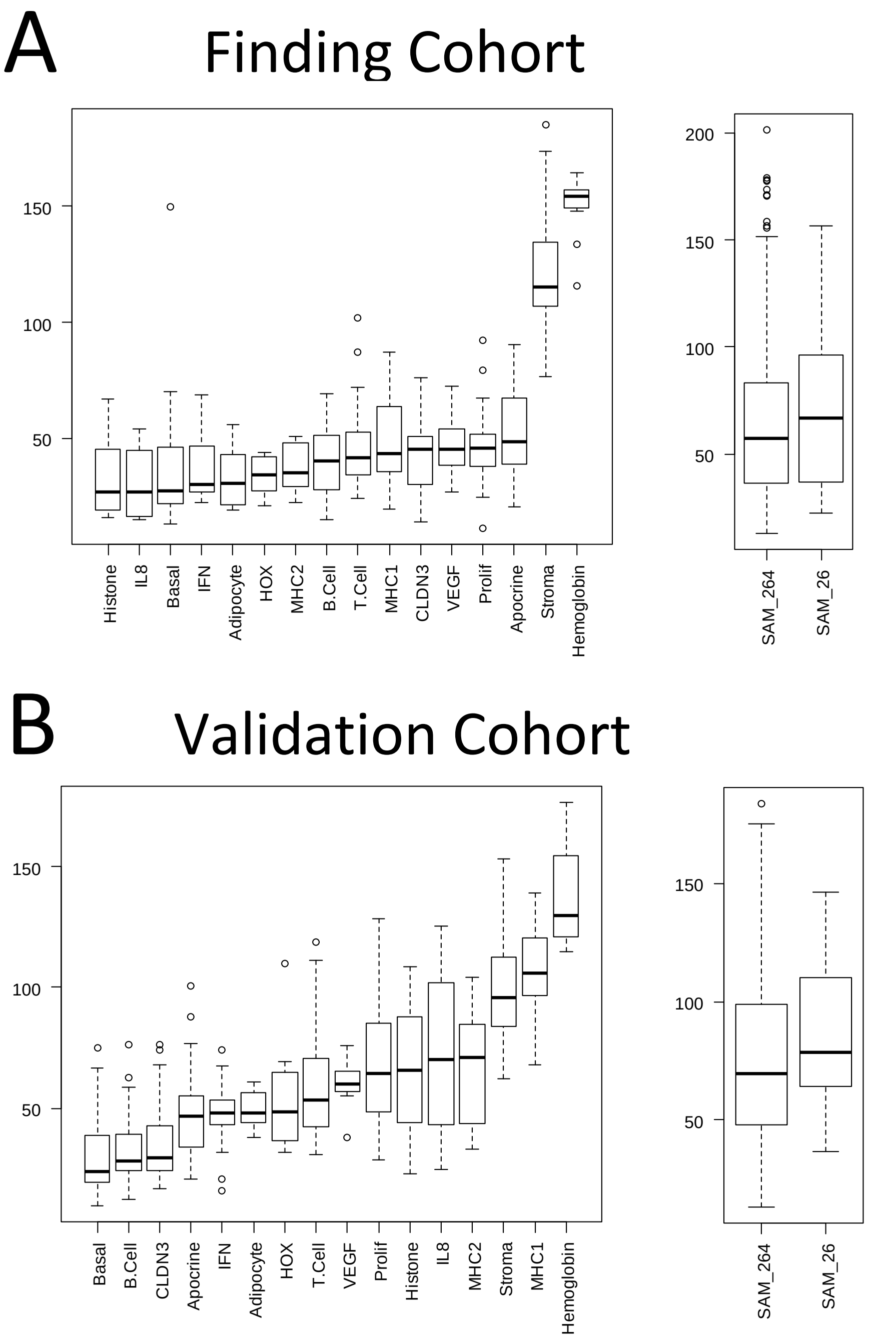

Supplementary Figure S5: Analysis of dataset bias of metagenes and the prognostic signatures

A) The dependence of earch probeset from the U133A array on the dataset vector was analyzed using the standard Kruskal-Wallis rank test in the finding cohort of 394 samples (see Suppl. Fig. S2). Box plots are shown for the Kruskal-Wallis statistics of the probesets of each metagene on the left and for the two prognostic signatures on the right. The highest dataset bias was observed for Stroma and Hemoglobin metagenes which is related to different applied biopsy methods (fine needle biopsy vs. surgical resection).
B) The 261 samples from the validation cohort were used to calculate the Kruskal-Wallis rank sum statistics for all probesets. Again box plots are shown as in (A), but the Kruskal-Wallis statistics from the validation cohort were applied. Several metagenes are characterized by higher bias in the validation cohort.
