# Supplementary material for: Homogeneous Datasets of Triple Negative Breast Cancers Enable the Identification of Novel Prognostic and Predictive Signatures
Source: PLoS One. 2011 Dec 29;6(12):e28403. doi: 10.1371/journal.pone.0028403 (PMC3248403; doi:10.1371/journal.pone.0028403)
Supplement: Figure S6 — Correlation of individual markers from the prognostic signatures with known metagenes in triple negative breast cancer. From the 264 Affymetrix probsets of the supervised prognostic signature, 235 probesets were associated with poor prognosis (analyzed in panels A and C) and 29 with good prognosis (analyzed in panels B and D). A) The 235 individual probesets associated with poor prognosis (horizontically) were analyzed for their correlation with the expression of 16 metagenes (vertically) for molecular phenotypes in the 394 TNBC samples from the finding cohort. 116 probesets displaying a Pearson correlation above a cutoff 0.2 are sorted (horizontically) on the left according to the assigned metagene while 60 probesets remained unclassified. B) The 29 individual probesets associated with good prognosis were analyzed as in (A) and 21 assigned to metagenes (cutoff 0.2) are sorted horizontically on the left while 8 remained unclassified. C) The same analysis as in (A) was performed using the more stringent cutoff 0.3 for assignment to a metagene resulting in 118 probesets correlated to metagenes from the list of 235 probesets associated with poor prognosis. D) The same analysis as in (B) was perfomed using the more stringent cutoff 0.3 resulting in 18 of the 29 good prognosis probesets assigned to metagenes. All individual correlation values are given in Supplementary Table S3. (PDF) [file pone.0028403.s006.pdf]

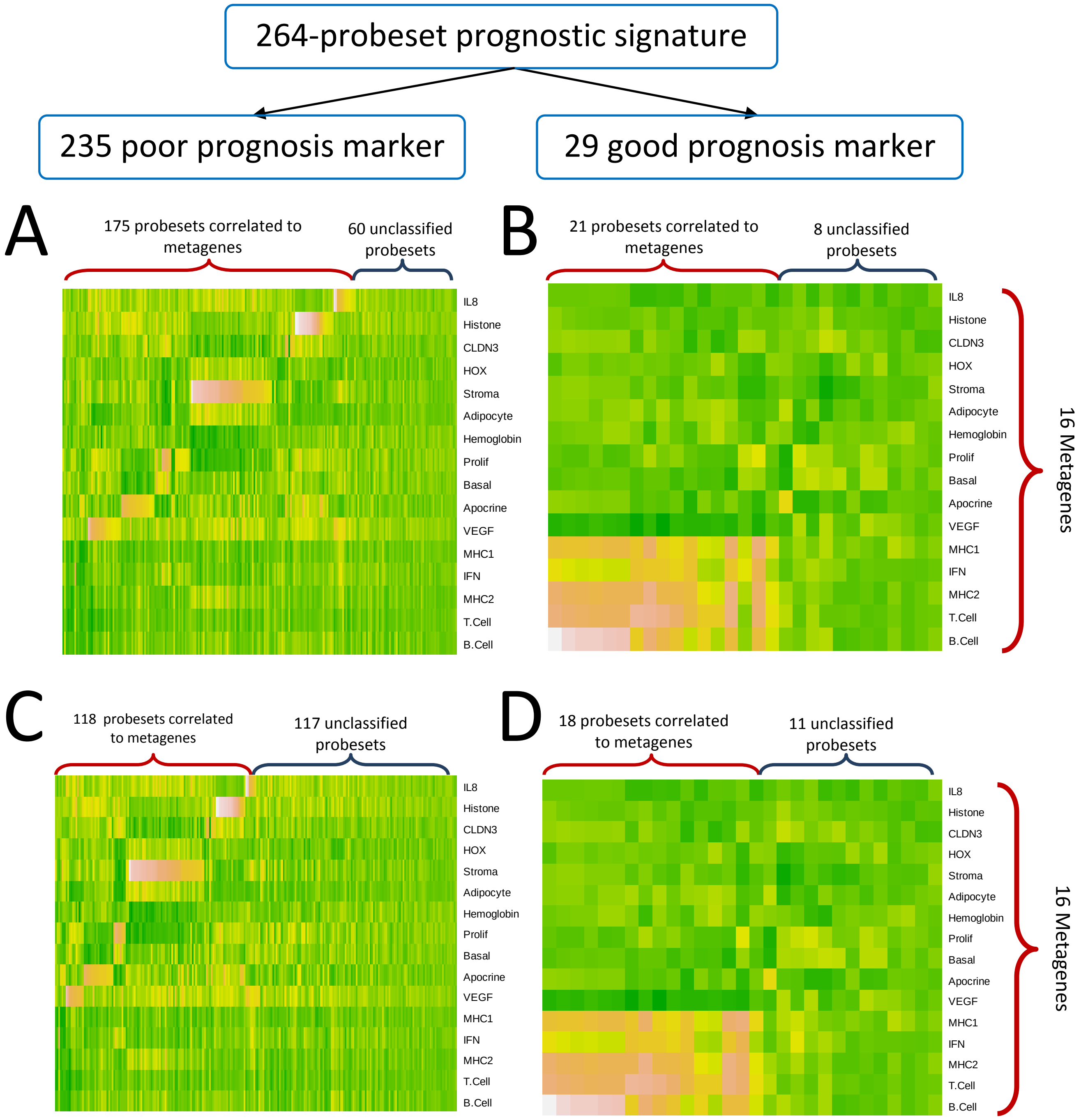

Supplementary Figure S6: Correlation of individual markers from the prognostic signatures with known metagenes in triple negative breast cancer

From the 264 Affymetrix probsets of the supervised prognostic signature, 235 probesets were associated with poor prognosis (analyzed in panels A and C) and 29 with good prognosis (analyzed in panels B and D).

A) The 235 individual probesets associated with poor prognosis (horizontically) were analyzed for their correlation with the expression of 16 metagenes (vertically) for molecular phenotypes in the 394 TNBC samples from the finding cohort. 116 probesets displaying a Pearson correlation above a cutoff 0.2 are sorted (horizontically) on the left according to the assigned metagene while 60 probesets remained unclassified.

B) The 29 individual probesets associated with good prognosis were analyzed as in (*A*) and 21 assigned to metagenes (cutoff 0.2) are sorted horizontically on the left while 8 remained unclassified.

C) The same analysis as in (*A*) was performed using the more stringent cutoff 0.3 for assignment to a metagene resulting in 118 probesets correlated to metagenes from the list of 235 probesets associated with poor prognosis.

D) The same analysis as in (*B*) was perfomed using the more stringent cutoff 0.3 resulting in 18 of the 29 good prognosis probesets assigned to metagenes.

All individual correlation values are given in Supplementary Table S3.
