# Supplementary material for: Homogeneous Datasets of Triple Negative Breast Cancers Enable the Identification of Novel Prognostic and Predictive Signatures
Source: PLoS One. 2011 Dec 29;6(12):e28403. doi: 10.1371/journal.pone.0028403 (PMC3248403; doi:10.1371/journal.pone.0028403)
Supplement: Figure S7 — Relationship of the 26 probeset signature to the 16 metagenes and seven known prognostic signatures in TNBC. The 394 TNBC samples were analyzed for the expression of 16 metagenes and seven previously published prognostic signatures as described in Figure 3 and hierachical clustered using Pearson correlation as distance metric. Abbreviations: SAMmean = 26 probeset signature wound.score$score = Wound response signature rs.394$score = recurrence score ggi.score$score = genomic grade index gene70.score$score = 70-gene signature sabatier.score$score = medullary like signature Tesch7.score$score = 7-gene immune response module sdpp.sore$score = stroma derived prognostic predictor. (PDF) [file pone.0028403.s007.pdf]

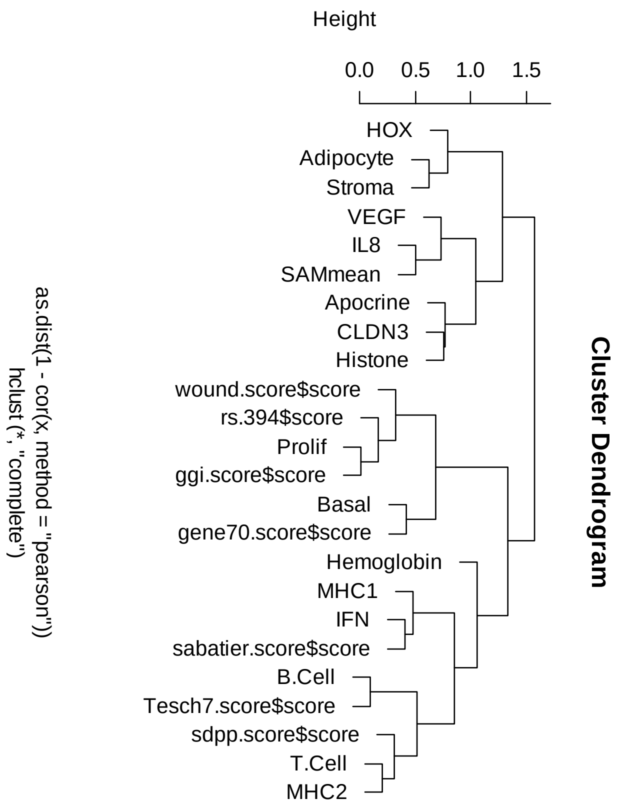

Supplementary Figure S7: Relationship of the 26 probeset signature to the 16 metagenes and seven known prognostic signatures in TNBC

The 394 TNBC samples were analyzed for the expression of 16 metagenes and seven previously published prognostic signatures as described in Figure 3 and hierachical clustered using Pearson correlation as distance metric.

Abbreviations:

`SAMmean` = 26 probeset signature
`wound.score$score` = Wound response signature
`rs.394$score` = recurrence score
`ggi.score$score` = genomic grade index
`gene70.score$score` = 70-gene signature
`sabatier.score$score` = medullary like signature
`Tesch7.score$score` = 7-gene immune response module
`sdpp.sore$score` = stroma derived prognostic predictor
