# Supplementary material for: Homogeneous Datasets of Triple Negative Breast Cancers Enable the Identification of Novel Prognostic and Predictive Signatures
Source: PLoS One. 2011 Dec 29;6(12):e28403. doi: 10.1371/journal.pone.0028403 (PMC3248403; doi:10.1371/journal.pone.0028403)
Supplement: Figure S8 — Stability analysis of the prognostic signatures from the supervised analysis. The 264 Affymetrix probsets of the supervised prognostic signature were filtered according to their dataset bias measured through Kruskal-Wallis statistic and different stringency from SAM analysis as given in the Table below the graphs. The resulting probeset lists of 252, 24, 181, and 16 probesets were used for prognostic signature generation as the original 264 probeset list. In upper panels A, B, C, and D the correlation of the four alternative signatures to the 264-probeset signature is shown by scatter plot analysis. The lower panels display the results from the Kaplan-Meier analyses of the validation cohort of 261 TNBC (105 samples with follow up information). In addition P-Values of multivariate Cox regression analysis of the validation cohort using continous signature scores are given in the table below. (PDF) [file pone.0028403.s008.pdf]

Panel:	A	B	C	D
Number of probesets:	252	24	181	16
Correlation to 264-probeset signature:	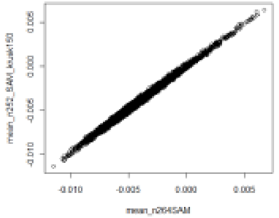	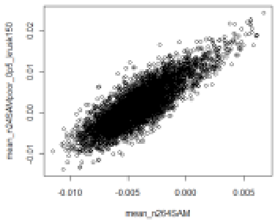	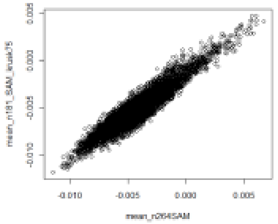	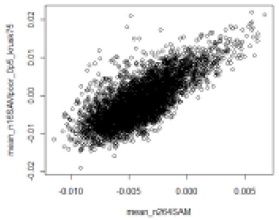
Kaplan-Meier analysis of highest quartile in validation cohort:	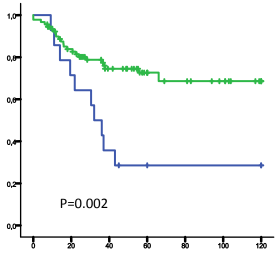	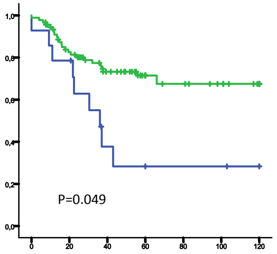	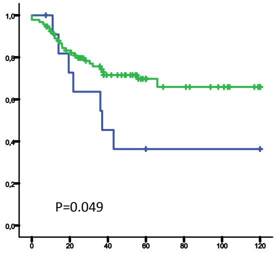	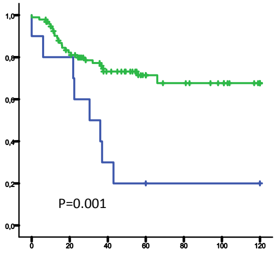

	Filtering criteria			Survival analysis in validation cohort	
**Figure panel**	**Dataset bias cutoff (Kruskal-stat)**	**Stringency / FDR (%)**	**Number of resulting probesets**	**Kaplan-Meier analysis of highest quartile**	**Continous score in multivariate Cox regression**
(Fig. 2B)	none	Low / 25 %	264	P=0.009	P=0.001
(Fig. 2D)	none	High / <3.5 %	26	P=0.001	P=0.001
**A**	<150	Low / 25 %	252	P=0.002	P=0.002
**B**	<150	High / <3.5 %	24	P=0.005	P=0.011
**C**	<75	Low / 25 %	181	P=0.049	P=0.004
**D**	<75	High / <3.5 %	16	P=0.001	P=0.024

Supplementary Figure S8: Stability analysis of the prognostic signatures from the supervised analysis

The 264 Affymetrix probsets of the supervised prognostic signature were filtered according to their dataset bias measured through Kruskal-Wallis statistic and different stringency from SAM analysis as given in the Table below the graphs. The resulting probeset lists of 252, 24, 181, and 16 probesets were used for prognostic signature generation as the original 264 probeset list. In upper panels A, B, C, and D the correlation of the four alternative signatures to the 264-probeset signature is shown by scatter plot analysis. The lower panels display the results from the Kaplan-Meier analyses of the validation cohort of 261 TNBC (105 samples with follow up information). In addition P-Values of multivariate Cox regression analysis of the validation cohort using continous signature scores are given in the table below.
