# Supplementary material for: Homogeneous Datasets of Triple Negative Breast Cancers Enable the Identification of Novel Prognostic and Predictive Signatures
Source: PLoS One. 2011 Dec 29;6(12):e28403. doi: 10.1371/journal.pone.0028403 (PMC3248403; doi:10.1371/journal.pone.0028403)
Supplement: Figure S9 — Expression of the 264-probeset and 26-probeset signature scores in seven different TNBC subtypes according to Lehmann et al. A) Box plots comparing the expression of the 264-probeset signature in the seven different TNBC subtypes according to Lehmann et al. (J Clin Invest. 2011; 121: 2750) separately for our finding and validation cohorts. No clear correlation of the expression of the signature with any of the subtypes was observed. The seven subtypes have been ordered according the expression of the signature in the finding cohort. Highest expression was observed in the “basal-like 2” (BL2) and “luminal androgen receptor” (LAR) subtypes of the finding cohort. However this effect was not reproduced in the validation cohort. B) The same analysis as in (A) was performed for the expression of the 26-probeset signature. The observed result was similar in that no reproducible correlation of the signature with any subtypes was detected. (PDF) [file pone.0028403.s009.pdf]

A

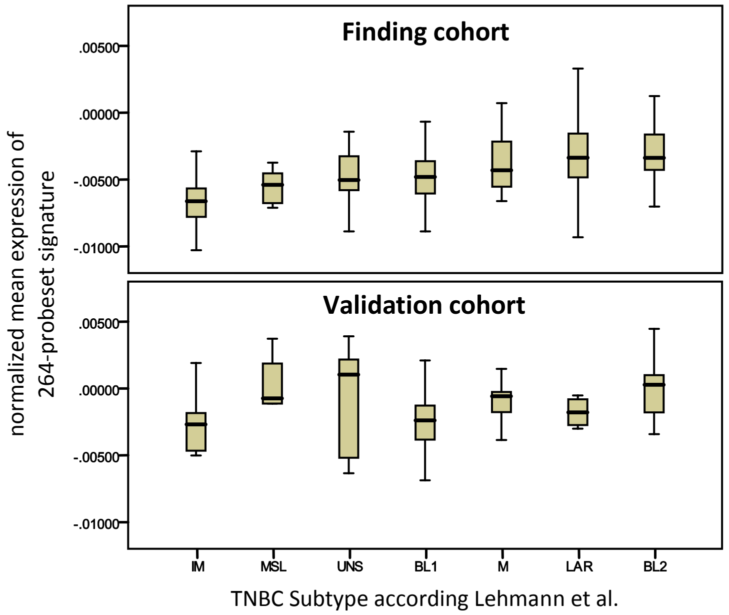

B

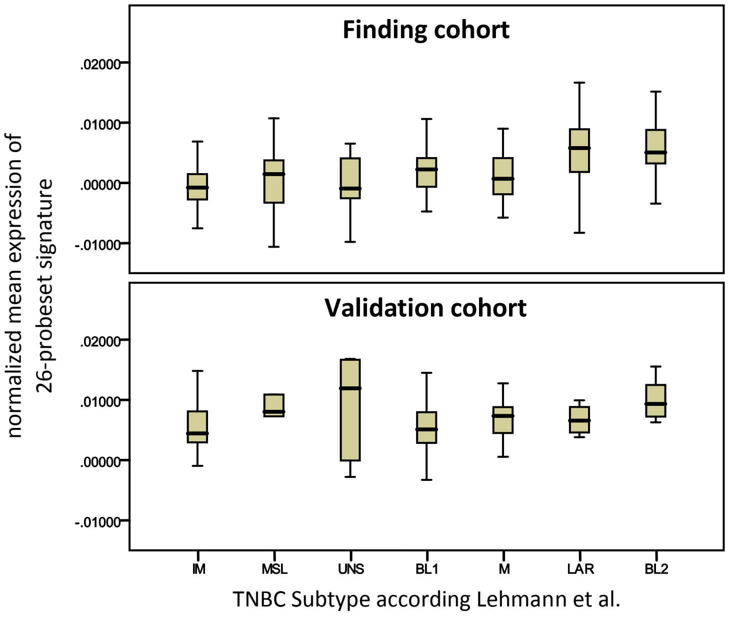

Supplementary Figure S9: **Expression of the 264-probeset and 26-probeset signature scores in seven different TNBC subtypes according to Lehmann et al.**

A) Box plots comparing the expression of the 264-probeset signature in the seven different TNBC subtypes according to Lehmann et al. (J Clin Invest. 2011; 121: 2750) separately for our finding and validation cohorts.
No clear correlation of the expression of the signature with any of the subtypes was observed. The seven subtypes have been ordered according the expression of the signature in the finding cohort. Highest expression was observed in the "basal-like 2" (BL2) and "luminal androgen receptor" (LAR) subtypes of the finding cohort. However this effect was not reproduced in the validation cohort.

B) The same analysis as in (*A*) was performed for the expression of the 26-probeset signature. The observed result was similar in that no reproducible correlation of the signature with any subtypes was detected.
