# Supplementary material for: Homogeneous Datasets of Triple Negative Breast Cancers Enable the Identification of Novel Prognostic and Predictive Signatures
Source: PLoS One. 2011 Dec 29;6(12):e28403. doi: 10.1371/journal.pone.0028403 (PMC3248403; doi:10.1371/journal.pone.0028403)
Supplement: Table S1 — Summary of Affymetrix microarray datasets used in this study. (PDF) [file pone.0028403.s010.pdf]

Supplementary Table S1: Summary of Affymetrix microarray datasets used in this study

*Dataset **	*Data Source*	*% of samples (complete datasets)*								*No. of samples*							
		Age ≤50	*Tumor size ≤2cm*	*LNN*	*G3*	*No systemic treatment*	*endocrine treatment*	*chemotherapy*	*Relapse*	*total*	*TNBC*	*Finding cohort*	*Validation cohort*	*Dataset Comparability metric C*	*DMFS data only*	*surgical biopsies*	*Reference*
Rotterdam-EMC344	*GSE2034, GSE5327*	*55*	*35*	*100*	*72*	*100*	*0*	*0*	*28*	*344*	*82*	*82*	*0*	*0.0085*	*x*	*+*	*i,ii,iii*
TransBIG	*GSE7390*	*80*	*18*	*100*	*75*	*100*	*0*	*0*	*43*	*198*	*40*	*40*	*0*	*0.0110*		*+*	*iv*
Mainz	*GSE11121*	*33*	*33*	*100*	*71*	*100*	*0*	*0*	*38*	*200*	*21*	*21*	*0*	*0.0115*	*x*	*+*	*v*
Stockholm	*GSE1456*				*74*	*45*	*55*	*0*	*40*	*159*	*25*	*25*	*0*	*0.0119*		*+*	*vi*
Uppsala	*GSE3494 (n=251), GSE6232 (n=5), GSE4922 (n=1), GSE2990 (n=1)*	*37*	*41*	*68*	*59*	*86*	*14*	*0*	*26*	*258*	*27*	*27*	*0*	*0.0124*		*+*	*vii,viii*
Frankfurt-2	*This study*	*50*	*0*	*39*	*56*				*33*	*67*	*19*	*19*	*0*	*0.0146*		*-*	*ix*
Frankfurt	*This study*	*33*	*42*	*83*	*83*	*0*	*0*	*100*	*33*	*119*	*24*	*24*	*0*	*0.0155*		*+*	*x*
New York	*GSE2603*	*40*	*11*	*49*		*0*	*4*	*96*	*48*	*99*	*35*	*35*	*0*	*0.0155*	*x*	*+*	*xi*
Oxford-Untreated	*GSE2990 (n=61), GSE6532 (n=8)*	*54*	*46*	*100*	*60*	*100*	*0*	*0*	*45*	*69*	*13*	*13*	*0*	*0.0157*		*+*	*xii*
Hamburg-2	*This study*	*0*	*0*	*71*	*40*	*0*	*100*	*0*	*20*	*77*	*7*	*7*	*0*	*0.0180*	*x*	*+*	*xiii*
Hamburg-1	*This study*	*53*	*33*	*80*	*86*	*0*	*0*	*100*	*47*	*77*	*15*	*15*	*0*	*0.0182*		*+*	*xiv*
Signapore	*GSE5364*									*183*	*36*	*36*	*0*	*0.0186*		*+*	*xv*
MDA133	*mdanderson.org*	*38*	*21*	*24*	*83*					*133*	*29*	*29*	*0*	*0.0189*		*-*	*xvi*
Boston	*GSE3744*				*100*					*40*	*16*	*16*	*0*	*0.0224*		*+*	*xvii*
Tampa	*GSE10780*									*39*	*5*	*5*	*0*	*0.0262*		*+*	*xviii*
Rotterdam-EMC204	*GSE12276*	*55*	*22*		*71*	*63*	*25*	*13*		*204*	*56*	*0*	*56*	*0.0320*		*+*	*xix*
MDA100	*GSE16716*	*48*	*3*	*18*	*73*	*0*	*0*	*100*		*100*	*33*	*0*	*33*	*0.0327*		*-*	*xx*
EORTC	*GSE1561*		*0*	*43*	*68*					*49*	*21*	*0*	*21*	*0.0338*		*-*	*xxi*
Genentech	*GSE12763*									*30*	*5*	*0*	*5*	*0.0389*		*+*	*xxii*
Frankfurt-3	*This study*	*0*	*50*	*50*	*50*	*0*	*100*	*0*	*50*	*52*	*2*	*0*	*2*	*0.0427*		*+*	*xxiii*
San Francisco	*E-TABM-158*	*52*	*35*	*52*	*45*	*17*	*13*	*70*	*22*	*118*	*23*	*0*	*23*	*0.0441*	*x*	*+*	*xxiv*
Paris	*GSE13787*				*100*					*23*	*10*	*0*	*10*	*0.0487*		*+*	*xxv*
Berlin	*GSE6596*	*29*	*29*		*71*					*24*	*7*	*0*	*7*	*0.0598*		*+*	*xxvi*
Veridex-Tam	*GSE12093*			*100*		*0*	*100*	*0*	*0*	*136*	*1*	*0*	*1*	*0.0650*	*x*	*+*	*xxvii*
London-2	*GSE9195*	*0*	*100*	*100*	*100*	*0*	*100*	*0*	*0*	*77*	*2*	*0*	*2*	*0.0663*		*+*	*xxviii*
London	*GSE6532*	*50*	*0*	*0*	*100*	*0*	*100*	*0*	*50*	*87*	*2*	*0*	*2*	*0.0687*		*+*	*xxix*
expO	*GSE2109*	*35*	*33*	*42*	*83*					*301*	*22*	*0*	*22*	*0.1001*		*+*	*xxx*
Edinburgh	*GSE5462*					*0*	*100*	*0*		*116*	*1*	*0*	*1*	*0.1285*		*-*	*xxxi*
Oxford-Tamoxifen	*GSE6532*									*109*	*0*			*n.a.*			*xii*
TOTAL:		***48***	***25***	***68***	***72***	***57***	***12***	***31***	***35***	***3488***	***579***	***394***	***185***			***495***	
Additional datasets:																	
**Validation-Cohort**	*GSE21653*									*266*	*76*	*0*	*76*			*+*	*xxxii*
**Total**												***394***	***261***				

**** Remarks**: Datasets are sorted according to the comparability of the microarrays of only TNBC samples. The complete TransBIG dataset contains indepen*dent replicate samples from 19 patients of the Uppsala cohort and 22 patients of the Oxford-Untreated cohort. Affymetrix HG-U133A microarrays were applied in all studies except that in part HG-U133Plus arrays were used in datasets GSE2109, GSE3744, GSE6532, GSE9195, GSE10780, GSE12763, GSE12276, and GSE13787. In these cases only the probe sets identical to HG-U133A arrays were used.

References for Supplementary Table S1:

[i] Wang Y, Klijn JG, Zhang Y, Sieuwerts AM, Look MP, Yang F, Talantov D, Timmermans M, Meijer-van Gelder ME, Yu J, Jatkoe T, Berns EM, Atkins D, Foekens JA. Gene-expression profiles to predict distant metastasis of lymph-node-negative primary breast cancer. Lancet. 2005; 365(9460):671-9.

[ii] Minn AJ, Gupta GP, Padua D, Bos P, Nguyen DX, Nuyten D, Kreike B, Zhang Y, Wang Y, Ishwaran H, Foekens JA, van de Vijver M, Massagué J. Lung metastasis genes couple breast tumor size and metastatic spread. Proc Natl Acad Sci U S A. 2007 Apr 17;104(16):6740-5.

[iii] Creighton CJ, Kent Osborne C, van de Vijver MJ, Foekens JA, Klijn JG, Horlings HM, Nuyten D, Wang Y, Zhang Y, Chamness GC, Hilsenbeck SG, Lee AV, Schiff R. Molecular profiles of progesterone receptor loss in human breast tumors. Breast Cancer Res Treat. 2009 Mar;114(2):287-99.

[iv] Desmedt C, Piette F, Loi S, Wang Y, Lallemand F, Haibe-Kains B, Viale G, Delorenzi M, Zhang Y, d'Assignies MS, Bergh J, Lidereau R, Ellis P, Harris AL, Klijn JG, Foekens JA, Cardoso F, Piccart MJ, Buyse M, Sotiriou C; TRANSBIG Consortium. Strong time dependence of the 76-gene prognostic signature for node-negative breast cancer patients in the TRANSBIG multicenter independent validation series. Clin Cancer Res. 2007 Jun 1;13(11):3207-14.

[v] Schmidt M, Böhm D, von Törne C, Steiner E, Puhl A, Pilch H, Lehr HA, Hengstler JG, Kölbl H, Gehrmann M. The humoral immune system has a key prognostic impact in node-negative breast cancer. Cancer Res. 2008 Jul 1;68(13):5405-13.

[vi] Pawitan Y, Bjohle J, Amler L, Borg AL, Egyhazi S, Hall P, Han X, Holmberg L, Huang F, Klaar S, Liu ET, Miller L, Nordgren H, Ploner A, Sandelin K, Shaw PM, Smeds J, Skoog L, Wedren S, Bergh J. Gene expression profiling spares early breast cancer patients from adjuvant therapy: derived and validated in two population-based cohorts. Breast Cancer Res. 2005;7(6):R953-64.

[vii] Miller LD, Smeds J, George J, Vega VB, Vergara L, Ploner A, Pawitan Y, Hall P, Klaar S, Liu ET, Bergh J. An expression signature for p53 status in human breast cancer predicts mutation status, transcriptional effects, and patient survival. Proc Natl Acad Sci U S A. 2005 Sep 20;102(38):13550-5.

[viii] Ivshina AV, George J, Senko O, Mow B, Putti T, Smeds J, Lindahl T, Pawitan Y, Hall P, Nordgren H, Wong, John EL, Liu ET, Bergh J, Kuznetsov VA, Miller LD. Genetic reclassification of histologic grade delineates new clinical subtypes of breast cancer. Cancer Res. 2006 Nov 1;66(21):10292-301.

[ix] Rody A, Karn T, Solbach C, Gaetje R, Munnes M, Kissler S, Ruckhäberle E, Minckwitz GV, Loibl S, Holtrich U, Kaufmann M. The erbB2+ cluster of the intrinsic gene set predicts tumor response of breast cancer patients receiving neoadjuvant chemotherapy with docetaxel, doxorubicin and cyclophosphamide within the GEPARTRIO trial. Breast. 2007 Jun;16(3):235-40.

[x] Rody A, Holtrich U, Gaetje R, Gehrmann M, Engels K, von Minckwitz G, Loibl S, Diallo-Danebrock R, Ruckhäberle E, Metzler D, Ahr A, Solbach C, Karn T, Kaufmann M. Poor outcome in estrogen receptor-positive breast cancers predicted by loss of plexin B1. Clin Cancer Res. 2007 Feb 15;13(4):1115-22.

[xi] Minn AJ, Gupta GP, Siegel PM, Bos PD, Shu W, Giri DD, Viale A, Olshen AB, Gerald WL, Massagué J. Genes that mediate breast cancer metastasis to lung. Nature. 2005 Jul 28;436(7050):518-24.

[xii] Sotiriou C, Wirapati P, Loi S, Harris A, Fox S, Smeds J, Nordgren H, Farmer P, Praz V, Haibe-Kains B, Desmedt C, Larsimont D, Cardoso F, Peterse H, Nuyten D, Buyse M, Van de Vijver MJ, Bergh J, Piccart M, Delorenzi M. Gene expression profiling in breast cancer: understanding the molecular basis of histologic grade to improve prognosis. J Natl Cancer Inst. 2006 Feb 15;98(4):262-72.

[xiii] Rody A, Holtrich U, Muller V, et al. c-Kit: identification of coregulated genes by gene expression profiling and clinical relevance of two breast cancer subtypes with stem cell like features. 2006. ASCO Annual Meeting Proceedings Part I. J Clin Oncol 2006;24:622.

[xiv] Rody A, Karn T, Ruckhäberle E, Hanker L, Metzler D, Müller V, Solbach C, Ahr A, Gätje R, Holtrich U, Kaufmann M. Loss of Plexin B1 is highly prognostic in low proliferating ER positive breast cancers--results of a large scale microarray analysis. Eur J Cancer. 2009 Feb;45(3):405-13.

[xv] Yu K, Ganesan K, Tan LK, Laban M, Wu J, Zhao XD, Li H, Leung CH, Zhu Y, Wei CL, Hooi SC, Miller L, Tan P. A precisely regulated gene expression cassette potently modulates metastasis and survival in multiple solid cancers. PLoS Genet. 2008 Jul 18;4(7):e1000129.

[xvi] Hess KR, Anderson K, Symmans WF, Valero V, Ibrahim N, Mejia JA, Booser D, Theriault RL, Buzdar AU, Dempsey PJ, Rouzier R, Sneige N, Ross JS, Vidaurre T, Gómez HL, Hortobagyi GN, Pusztai L. Pharmacogenomic predictor of sensitivity to preoperative chemotherapy with paclitaxel and fluorouracil, doxorubicin, and cyclophosphamide in breast cancer. J Clin Oncol. 2006 Sep 10;24(26):4236-44.

[xvii] Richardson AL, Wang ZC, De Nicolo A, Lu X, Brown M, Miron A, Liao X, Iglehart JD, Livingston DM, Ganesan S. X chromosomal abnormalities in basal-like human breast cancer. Cancer Cell. 2006 Feb;9(2):121-32.

[xviii] Chen DT, Nasir A, Culhane A, Venkataramu C, Fulp W, Rubio R, Wang T, Agrawal D, McCarthy SM, Gruidl M, Bloom G, Anderson T, White J, Quackenbush J, Yeatman T. Proliferative genes dominate malignancy-risk gene signature in histologically-normal breast tissue. Breast Cancer Res Treat. 2009 Mar 6.

[xix] Bos PD, Zhang XH, Nadal C, Shu W, Gomis RR, Nguyen DX, Minn AJ, van de Vijver MJ, Gerald WL, Foekens JA, Massagué J. Genes that mediate breast cancer metastasis to the brain. Nature. 2009 Jun 18;459(7249):1005-9.

[xx] Popovici V, Chen W, Gallas BG, Hatzis C, Shi W, Samuelson FW, Nikolsky Y, Tsyganova M, Ishkin A, Nikolskaya T, Hess KR, Valero V, Booser D, Delorenzi M, Hortobagyi GN, Shi L, Symmans WF, Pusztai L. Effect of training sample size and classification difficulty on the accuracy of genomic predictors. Breast Cancer Res. 2010 Jan 11;12(1):R5.

[xxi] Farmer P, Bonnefoi H, Becette V, Tubiana-Hulin M, Fumoleau P, Larsimont D, Macgrogan G, Bergh J, Cameron D, Goldstein D, Duss S, Nicoulaz AL, Brisken C, Fiche M, Delorenzi M, Iggo R. Identification of molecular apocrine breast tumours by microarray analysis. Oncogene. 2005 Jul 7;24(29):4660-71.

[xxii] Gene Expression Omnibus. Series GSE12763 http://www.ncbi.nlm.nih.gov/geo/query/acc.cgi?acc=GSE12763

[xxiii] Ruckhäberle E, Rody A, Engels K, Gaetje R, von Minckwitz G, Schiffmann S, Grösch S, Geisslinger G, Holtrich U, Karn T, Kaufmann M. Microarray analysis of altered sphingolipid metabolism reveals prognostic significance of sphingosine kinase 1 in breast cancer. Breast Cancer Res Treat. 2008 Nov;112(1):41-52.

[xxiv] Chin K, DeVries S, Fridlyand J, Spellman PT, Roydasgupta R, Kuo WL, Lapuk A, Neve RM, Qian Z, Ryder T, Chen F, Feiler H, Tokuyasu T, Kingsley C, Dairkee S, Meng Z, Chew K, Pinkel D, Jain A, Ljung BM, Esserman L, Albertson DG, Waldman FM, Gray JW. Genomic and transcriptional aberrations linked to breast cancer pathophysiologies. Cancer Cell. 2006 Dec;10(6):529-41.

[xxv] Marty B, Maire V, Gravier E, Rigaill G, Vincent-Salomon A, Kappler M, Lebigot I, Djelti F, Tourdès A, Gestraud P, Hupé P, Barillot E, Cruzalegui F, Tucker GC, Stern MH, Thiery JP, Hickman JA, Dubois T. Frequent PTEN genomic alterations and activated phosphatidylinositol 3-kinase pathway in basal-like breast cancer cells. Breast Cancer Res. 2008;10(6):R101. Epub 2008 Dec 3.

[xxvi] Klein A, Wessel R, Graessmann M, Jürgens M, Petersen I, Schmutzler R, Niederacher D, Arnold N, Meindl A, Scherneck S, Seitz S, Graessmann A. Comparison of gene expression data from human and mouse breast cancers: identification of a conserved breast tumor gene set. Int J Cancer. 2007 Aug 1;121(3):683-8.

[xxvii] Zhang Y, Sieuwerts AM, McGreevy M, Casey G, Cufer T, Paradiso A, Harbeck N, Span PN, Hicks DG, Crowe J, Tubbs RR, Budd GT, Lyons J, Sweep FC, Schmitt M, Schittulli F, Golouh R, Talantov D, Wang Y, Foekens JA. The 76-gene signature defines high-risk patients that benefit from adjuvant tamoxifen therapy. Breast Cancer Res Treat. 2008 Sep 23. [Epub ahead of print]

[xxviii] Loi S, Haibe-Kains B, Desmedt C, Wirapati P, Lallemand F, Tutt AM, Gillet C, Ellis P, Ryder K, Reid JF, Daidone MG, Pierotti MA, Berns EM, Jansen MP, Foekens JA, Delorenzi M, Bontempi G, Piccart MJ, Sotiriou C. Predicting prognosis using molecular profiling in estrogen receptor-positive breast cancer treated with tamoxifen. BMC Genomics. 2008 May 22;9:239.

[xxix] Loi S, Haibe-Kains B, Desmedt C, Lallemand F, Tutt AM, Gillet C, Ellis P, Harris A, Bergh J, Foekens JA, Klijn JG, Larsimont D, Buyse M, Bontempi G, Delorenzi M, Piccart MJ, Sotiriou C. Definition of clinically distinct molecular subtypes in estrogen receptor-positive breast carcinomas through genomic grade. J Clin Oncol. 2007 Apr 1;25(10):1239-46.

[xxx] The International Genomics Consortium (IGC). The expO project (Expression Project For Oncology) http://www.intgen.org/

[xxxi] Miller WR, Larionov AA, Renshaw L, Anderson TJ, White S, Murray J, Murray E, Hampton G, Walker JR, Ho S, Krause A, Evans DB, Dixon JM. Changes in breast cancer transcriptional profiles after treatment with the aromatase inhibitor, letrozole. Pharmacogenet Genomics. 2007 Oct;17(10):813-26.

[xxxii] Sabatier R, Finetti P, Cervera N, Lambaudie E, Esterni B, Mamessier E, Tallet A, Chabannon C, Extra JM, Jacquemier J, Viens P, Birnbaum D, Bertucci F. A gene expression signature identifies two prognostic subgroups of basal breast cancer. Breast Cancer Res Treat. 2010 May 21. [Epub ahead of print]
