# Supplementary material for: Homogeneous Datasets of Triple Negative Breast Cancers Enable the Identification of Novel Prognostic and Predictive Signatures
Source: PLoS One. 2011 Dec 29;6(12):e28403. doi: 10.1371/journal.pone.0028403 (PMC3248403; doi:10.1371/journal.pone.0028403)
Supplement: Table S2 — List of 355 Affymetrix probesets used for metagene calculation. (PDF) [file pone.0028403.s011.pdf]

Supplementary Table S2:

List of 355 Affymetrix probe sets used for metagene calculation:

Metagene	Affymetrix ProbeSet	GeneSymbol	DatasetBias KruskalFindCohort
IL-8	204470_at	CXCL1	14.8
IL-8	202859_x_at	IL8	35.9
IL-8	211506_s_at	IL8	54
IL-8	209774_x_at	CXCL2	18.1
VEGF	200632_s_at	NDRG1	40
VEGF	210513_s_at	VEGF	46.8
VEGF	211527_x_at	VEGF	72.4
VEGF	210512_s_at	VEGF	45.2
VEGF	212171_x_at	VEGF	60.8
VEGF	202912_at	ADM	26.8
VEGF	221009_s_at	ANGPTL4	36.8
Proliferation	206102_at	KIAA0186	50.1
Proliferation	209172_s_at	CENPF	39.5
Proliferation	203418_at	CCNA2	42.7
Proliferation	204026_s_at	ZWINT	42.2
Proliferation	203213_at	CDC2	56.6
Proliferation	203214_x_at	CDC2	47.6
Proliferation	210559_s_at	CDC2	45.6
Proliferation	204170_s_at	CKS2	79.1
Proliferation	204092_s_at	STK6	55
Proliferation	208079_s_at	STK6	38.9
Proliferation	203362_s_at	MAD2L1	43.9
Proliferation	213226_at	CCNA2	59.5
Proliferation	202095_s_at	BIRC5	61.3
Proliferation	202613_at	CTPS	49
Proliferation	201291_s_at	TOP2A	92.1
Proliferation	201292_at	TOP2A	31.5
Proliferation	222039_at	LOC146909	49.7
Proliferation	204822_at	TTK	52.2
Proliferation	202954_at	UBE2C	31.8
Proliferation	203755_at	BUB1B	32.3
Proliferation	202705_at	CCNB2	35.5
Proliferation	204962_s_at	CENPA	65.2
Proliferation	209642_at	BUB1	46.5
Proliferation	202870_s_at	CDC20	44.2
Proliferation	209408_at	KIF2C	46.5
Proliferation	221520_s_at	CDCA8	50.7
Proliferation	218039_at	NUSAP1	24.5
Proliferation	206364_at	KIF14	47.2
Proliferation	204641_at	NEK2	28
Proliferation	207828_s_at	CENPF	43.6
Proliferation	219918_s_at	ASPM	36.8
Proliferation	204444_at	KIF11	45
Proliferation	218542_at	C10orf3	44.5
Proliferation	213008_at	FLJ10719	67.4
Proliferation	213007_at	FLJ10719	43.9
Proliferation	218009_s_at	PRC1	43.3
Proliferation	210052_s_at	TPX2	11.3
Proliferation	203764_at	DLG7	53
Proliferation	214710_s_at	CCNB1	49
Proliferation	218355_at	KIF4A	60
Proliferation	202580_x_at	FOXM1	35.4
Proliferation	221436_s_at	CDCA3	31.2
Proliferation	218755_at	KIF20A	24.8
Proliferation	218663_at	HCAP-G	49.2
Proliferation	219148_at	PBK	51.1
Proliferation	218585_s_at	RAMP	36.8
Proliferation	218726_at	DKFZp762E1312	52.2

(continued)

Metagene	Affymetrix ProbeSet	GeneSymbol	DatasetBias KruskalFindCohort
Basal-like	202341_s_at	TRIM2	149.9
Basal-like	202342_s_at	TRIM2	46
Basal-like	215945_s_at	TRIM2	65.5
Basal-like	203074_at	ANXA8	26
Basal-like	202504_at	TRIM29	18.4
Basal-like	211002_s_at	TRIM29	19.6
Basal-like	204268_at	S100A2	26.3
Basal-like	201820_at	KRT5	19.5
Basal-like	204855_at	SERPINB5	31.7
Basal-like	209351_at	KRT14	13.2
Basal-like	205157_s_at	KRT17	19
Basal-like	212236_x_at	KRT17	17.1
Basal-like	209800_at	KRT16	42.2
Basal-like	209126_x_at	KRT6B	26.7
Basal-like	213680_at	KRT6B	19.8
Basal-like	209125_at	KRT6A	26.6
Basal-like	214580_x_at	KRT6A	20.1
Basal-like	1438_at	EPHB3	55.6
Basal-like	204600_at	EPHB3	19.3
Basal-like	218176_at	MAGEF1	64.5
Basal-like	205044_at	GABRP	24.8
Basal-like	202035_s_at	SFRP1	70.3
Basal-like	202036_s_at	SFRP1	51.7
Basal-like	202037_s_at	SFRP1	38.2
Basal-like	209842_at	SOX10	33.6
Basal-like	220425_x_at	ROPN1	39.6
Basal-like	206560_s_at	MIA	47.4
Basal-like	209843_s_at	SOX10	64.4
Basal-like	220624_s_at	ELF5	52.1
Basal-like	220625_s_at	ELF5	38.1
Basal-like	212730_at	DMN	33.5
Basal-like	219615_s_at	KCNK5	21.8
Basal-like	209504_s_at	PLEKHB1	25.3
Basal-like	213260_at	FOXC1	26.7
Basal-like	218963_s_at	KRT23	27.2
Basal-like	205487_s_at	VGLL1	25.3
Basal-like	215729_s_at	VGLL1	30.5
Claudin-CD24	201650_at	KRT19	34.3
Claudin-CD24	201596_x_at	KRT18	29.7
Claudin-CD24	209008_x_at	KRT8	20.4
Claudin-CD24	209016_s_at	KRT7	25.7
Claudin-CD24	205980_s_at	ARHGAP8	76
Claudin-CD24	37117_at	ARHGAP8	52.6
Claudin-CD24	203953_s_at	CLDN3	54
Claudin-CD24	203954_x_at	CLDN3	30.8
Claudin-CD24	201428_at	CLDN4	44.2
Claudin-CD24	201839_s_at	TACSTD1	45.1
Claudin-CD24	218186_at	RAB25	16.1
Claudin-CD24	201510_at	ELF3	13.8
Claudin-CD24	210827_s_at	ELF3	46.7
Claudin-CD24	208650_s_at	CD24	48.9
Claudin-CD24	209772_s_at	CD24	47.8
Claudin-CD24	208651_x_at	CD24	40.9
Claudin-CD24	209771_x_at	CD24	71
Claudin-CD24	216379_x_at	CD24	70.1
Claudin-CD24	266_s_at	CD24	48.5
Apocrine	204941_s_at	ALDH3B2	76.4
Apocrine	204942_s_at	ALDH3B2	36.4
Apocrine	211110_s_at	AR	48.6
Apocrine	211621_at	AR	90.5
Apocrine	209173_at	AGR2	39
Apocrine	207131_x_at	GGT1	49.5
Apocrine	208284_x_at	GGT1	46.6
Apocrine	209919_x_at	GGT1	40.9
Apocrine	211417_x_at	GGT1	57.1
Apocrine	215603_x_at	GGT1	73.9
Apocrine	211416_x_at	GGTLA4	81.7
Apocrine	206463_s_at	DHRS2	57.4
Apocrine	214079_at	DHRS2	65.9
Apocrine	211682_x_at	UGT2B28	20.4
Apocrine	206714_at	ALOX15B	69.3
Apocrine	206509_at	PIP	47.3
Apocrine	204667_at	FOXA1	70.6
Apocrine	218211_s_at	MLPH	74.2
Apocrine	214451_at	TFAP2B	42.3
Apocrine	204607_at	HMGCS2	65.2
Apocrine	214243_s_at	SERHL	32.5
Apocrine	217276_x_at	dJ222E13.1	38.5
Apocrine	217284_x_at	dJ222E13.1	53.1
Apocrine	213441_x_at	SPDEF	48.2
Apocrine	214404_x_at	SPDEF	31.9
Apocrine	220192_x_at	SPDEF	22.8
Apocrine	215686_x_at	TFAP2B	30.1

(continued)

Metagene	Affymetrix ProbeSet	GeneSymbol	DatasetBias KruskalFindCohort
Histone	208583_x_at	HIST1H2AJ	55.5
Histone	208523_x_at	HIST1H2BI	44.7
Histone	209398_at	HIST1H1C	26.8
Histone	208180_s_at	H4FH	25.2
Histone	202708_s_at	HIST2H2BE	31.4
Histone	208546_x_at	HIST1H2BH	28.2
Histone	208490_x_at	HIST1H2BF	15.8
Histone	208527_x_at	HIST1H2BE	33.1
Histone	208579_x_at	H2BFS	54.9
Histone	209806_at	HIST1H2BK	20.3
Histone	209911_x_at	HIST1H2BD	46.3
Histone	222067_x_at	HIST1H2BD	17.1
Histone	214290_s_at	HIST2H2AA	17.1
Histone	218280_x_at	HIST2H2AA	18.1
Histone	215071_s_at	HIST1H2AC	18
Histone	210387_at	HIST1H2BG	66.7
Histone	215779_s_at	HIST1H2BG	56.5
Histone	214469_at	HIST1H2AE	21.1
Histone	214455_at	H2BFL	25.6
Adipocyte	203980_at	FABP4	21
Adipocyte	205913_at	PLIN	19.2
Adipocyte	207175_at	ADIPOQ	21.9
Adipocyte	209612_s_at	ADH1B	43.5
Adipocyte	209613_s_at	ADH1B	42.6
Adipocyte	206488_s_at	CD36	36.1
Adipocyte	209555_s_at	CD36	24.6
Adipocyte	209763_at	CHRDL1	55.8
Stroma	202766_s_at	FBN1	114.7
Stroma	207172_s_at	CDH11	127.4
Stroma	207173_x_at	CDH11	139.1
Stroma	200665_s_at	SPARC	115.1
Stroma	202465_at	PCOLCE	151.3
Stroma	201185_at	PRSS11	119.3
Stroma	201069_at	MMP2	130.7
Stroma	202273_at	PDGFRB	170.7
Stroma	204114_at	NID2	136
Stroma	201792_at	AEBP1	136.4
Stroma	201744_s_at	LUM	76.5
Stroma	201438_at	COL6A3	153.1
Stroma	202310_s_at	COL1A1	135.5
Stroma	202403_s_at	COL1A2	107.9
Stroma	202404_s_at	COL1A2	100.2
Stroma	201852_x_at	COL3A1	153.7
Stroma	215076_s_at	COL3A1	115.2
Stroma	211161_s_at	COL3A1	106.2
Stroma	221729_at	COL5A2	118.8
Stroma	221730_at	COL5A2	131.2
Stroma	202311_s_at	COL1A1	129.1
Stroma	203325_s_at	COL5A1	109.9
Stroma	212488_at	COL5A1	127.5
Stroma	212489_at	COL5A1	140
Stroma	210809_s_at	POSTN	86.2
Stroma	212667_at	SPARC	106.6
Stroma	209596_at	DKFZp564I1922	99.6
Stroma	209955_s_at	FAP	100
Stroma	201893_x_at	DCN	113.2
Stroma	211896_s_at	DCN	113.1
Stroma	211813_x_at	DCN	133.7
Stroma	209335_at	DCN	90
Stroma	213001_at	ANGPTL2	144.6
Stroma	213004_at	ANGPTL2	133.1
Stroma	208851_s_at	THY1	108.4
Stroma	213869_x_at	THY1	94.2
Stroma	213909_at	LRRC15	84.9
Stroma	204619_s_at	CSPG2	101.6
Stroma	204620_s_at	CSPG2	125.8
Stroma	221731_x_at	CSPG2	97.8
Stroma	221541_at	LCRISP2	111
Stroma	211571_s_at	CSPG2	185
Stroma	215646_s_at	CSPG2	173.6
Stroma	211719_x_at	FN1	113.2
Stroma	210495_x_at	FN1	121.8
Stroma	216442_x_at	FN1	113
Stroma	212464_s_at	FN1	107.1
IFN	202411_at	IFI27	22.2
IFN	202086_at	MX1	27
IFN	205483_s_at	G1P2	27.1
IFN	203153_at	IFIT1	29.8
IFN	204747_at	IFIT3	32.2
IFN	213797_at	RSAD2	44.9
IFN	204439_at	IFI44L	30.8
IFN	214453_s_at	IFI44	26
IFN	205552_s_at	OAS1	62
IFN	204972_at	OAS2	68.8
IFN	218400_at	OAS3	48.7
IFN	219352_at	HERC6	28.3
IFN	205660_at	OASL	28.5
IFN	210797_s_at	OASL	46.5

Metagene	Affymetrix ProbeSet	GeneSymbol	DatasetBias KruskalFindCohort
MHC-1	200905_x_at	HLA-E	49.9
MHC-1	217456_x_at	HLA-E	66.5
MHC-1	210514_x_at	HLA-G	20.6
MHC-1	209140_x_at	HLA-B	87.1
MHC-1	208812_x_at	HLA-B	79.9
MHC-1	216526_x_at	HLA-C	67.2
MHC-1	214459_x_at	HLA-C	53.7
MHC-1	211529_x_at	HLA-G	19.4
MHC-1	211528_x_at	HLA-G	43
MHC-1	208729_x_at	HLA-B	40.9
MHC-1	211911_x_at	HLA-B	43.3
MHC-1	217436_x_at	HLA-J	42
MHC-1	204806_x_at	HLA-F	21.7
MHC-1	221875_x_at	HLA-F	26.9
MHC-1	211799_x_at	HLA-A	35.8
MHC-1	213932_x_at	HLA-A	60.9
MHC-1	215313_x_at	HLA-A	63.9
T-Cell	209083_at	CORO1A	65.3
T-Cell	204891_s_at	LCK	44.9
T-Cell	206666_at	GZMK	36.2
T-Cell	201720_s_at	LAPTM5	102
T-Cell	201721_s_at	LAPTM5	46
T-Cell	204912_at	IL10RA	24.1
T-Cell	206150_at	TNFRSF7	49.1
T-Cell	204563_at	SELL	63.7
T-Cell	209670_at	TRA@	41.6
T-Cell	204118_at	CD48	40.3
T-Cell	205831_at	CD2	36.8
T-Cell	210915_x_at	TRBC1	36.9
T-Cell	213193_x_at	TRBC1	39.6
T-Cell	213539_at	CD3D	50.5
T-Cell	211796_s_at	TRBC1	46.9
T-Cell	211339_s_at	ITK	27.4
T-Cell	203416_at	CD53	30.6
T-Cell	211742_s_at	EVI2B	29.4
T-Cell	212588_at	PTPRC	31.2
T-Cell	209671_x_at	TRA@	72
T-Cell	210972_x_at	TRA@	71.2
T-Cell	211902_x_at	TRA@	87.3
T-Cell	220330_s_at	SAMSN1	32.7
T-Cell	38149_at	KIAA0053	42.6
T-Cell	219014_at	PLAC8	27.7
T-Cell	204661_at	CD52	54.7
T-Cell	34210_at	CD52	37.8
MHC-2	201137_s_at	HLA-DPB1	22.2
MHC-2	203932_at	HLA-DMB	38.9
MHC-2	209619_at	CD74	50.4
MHC-2	204670_x_at	HLA-DRB1	27.2
MHC-2	208306_x_at	HLA-DRB1	31.8
MHC-2	209312_x_at	HLA-DRB1	27.1
MHC-2	215193_x_at	HLA-DRB1	29.8
MHC-2	208894_at	HLA-DRA	35.5
MHC-2	210982_s_at	HLA-DRA	48.6
MHC-2	211991_s_at	HLA-DPA1	50.7
MHC-2	212671_s_at	HLA-DQA1	42
MHC-2	217478_s_at	HLA-DMA	35
MHC-2	212998_x_at	HLA-DQB1	48.1
MHC-2	211654_x_at	HLA-DQB1	29.1

(continued)

Metagene	Affymetrix ProbeSet	GeneSymbol	DatasetBias KruskalFindCohort
B-Cell	211639_x_at	IGHM	48.5
B-Cell	211633_x_at	IGHG1	65.6
B-Cell	211641_x_at	IGHG1	52.2
B-Cell	211634_x_at	IGHM	45.8
B-Cell	211635_x_at	IGHG3	44.1
B-Cell	211640_x_at	IGHG1	41.1
B-Cell	211798_x_at	IGLJ3	57.6
B-Cell	211881_x_at	IGLJ3	51.5
B-Cell	211637_x_at	LOC388078	51.2
B-Cell	216491_x_at	IGHM	54
B-Cell	211908_x_at	IGHG1	58.8
B-Cell	211650_x_at	IGHG1	46.4
B-Cell	216510_x_at	IGHG1	60.5
B-Cell	217281_x_at	IGHG1	60.3
B-Cell	211643_x_at	IGKC	32.5
B-Cell	213502_x_at	LOC91316	25.6
B-Cell	211430_s_at	IGH@	21.9
B-Cell	209138_x_at	IGLC2	18.1
B-Cell	214677_x_at	IGL@	18.1
B-Cell	215121_x_at	IGL@	21.3
B-Cell	215379_x_at	IGL@	15
B-Cell	221651_x_at	IGKC	28.2
B-Cell	221671_x_at	IGKC	27
B-Cell	211644_x_at	IGKC	36.9
B-Cell	214669_x_at	LOC440871	19.4
B-Cell	211645_x_at	IGKC	29.4
B-Cell	215176_x_at	IGKC	31.4
B-Cell	217378_x_at	LOC391427	35.1
B-Cell	217157_x_at	IGKC	35.3
B-Cell	214836_x_at	IGKC	20
B-Cell	216207_x_at	IGKV1D-13	27.8
B-Cell	217480_x_at	LOC339562	30.3
B-Cell	216576_x_at	- - -	32.6
B-Cell	216401_x_at	- - -	35.9
B-Cell	215946_x_at	IGLL1	30.5
B-Cell	214916_x_at	IGH@	46.3
B-Cell	216557_x_at	IGHG1	62.6
B-Cell	211868_x_at	IGHG1	46.5
B-Cell	216984_x_at	IGLJ3	46
B-Cell	217148_x_at	IGLC2	41
B-Cell	216542_x_at	MGC27165	68.6
B-Cell	214768_x_at	IGKC	27.7
B-Cell	214973_x_at	IGHD	54.8
B-Cell	217235_x_at	IGLJ3	44.3
B-Cell	215949_x_at	IGHM	69.1
B-Cell	214777_at	IGKC	39
B-Cell	217179_x_at	IGL@	26
Hemoglobin	204419_x_at	HBG2	133.7
Hemoglobin	204848_x_at	HBG1	115.9
Hemoglobin	209116_x_at	HBB	164.4
Hemoglobin	204018_x_at	HBA1	158.4
Hemoglobin	209458_x_at	HBA1	154.7
Hemoglobin	211745_x_at	HBA1	150.8
Hemoglobin	214414_x_at	HBA2	152
Hemoglobin	211696_x_at	HBB	155.4
Hemoglobin	217232_x_at	HBB	155.2
Hemoglobin	217414_x_at	HBA2	153.7
Hemoglobin	211699_x_at	HBA1	147.7
Hemoglobin	213515_x_at	HBG2	158.6
HOXA	206289_at	HOXA4	27.4
HOXA	206847_s_at	HOXA7	43.9
HOXA	209905_at	HOXA9	41.1
HOXA	214651_s_at	HOXA9	27.8
HOXA	213844_at	HOXA5	38.2
HOXA	213147_at	HOXA10	30.1
HOXA	213150_at	HOXA10	20.9
HOXA	213823_at	HOXA11	43
