# Supplementary material for: Homogeneous Datasets of Triple Negative Breast Cancers Enable the Identification of Novel Prognostic and Predictive Signatures
Source: PLoS One. 2011 Dec 29;6(12):e28403. doi: 10.1371/journal.pone.0028403 (PMC3248403; doi:10.1371/journal.pone.0028403)
Supplement: Table S4 — A) Multivariate Cox regression of continous 264-probeset signature and standard parameters for event free survival in the finding cohort B) Multivariate Cox regression of continous 26-probeset signature and standard parameters for event free survival in the finding cohort. (PDF) [file pone.0028403.s013.pdf]

Supplementary Table S4A: Multivariate Cox regression of continous 264-probeset signature and standard parameters for event free survival in the finding cohort

Metagene	B	SE	Wald statistic	P-Value*
**264-probeset signature (as continous variable)**	387.8	46.32	70.1	**<0.0001**
**lymph node status**	-0.34	0.332	1.04	0.31
**age (>50 vs ≤50)**	-0.43	0.237	3.33	0.068
**tumor size (≤1cm vs >1cm)**	-0.042	0.258	0.027	0.87
**Histol. grade (G3 vs G1&G2)**	0.14	0.260	0.27	0.60

* significant P-Values are given in bold

Supplementary Table S4B: Multivariate Cox regression of continous 26-probeset signature and standard parameters for event free survival in the finding cohort

Metagene	B	SE	Wald statistic	P-Value*
**26-probeset signature (as continous variable)**	156.0	23.07	45.7	**<0.0001**
**lymph node status**	-0.400	0.332	1.46	0.23
**age (>50 vs ≤50)**	-0.429	0.235	3.34	0.068
**tumor size (≤1cm vs >1cm)**	-0.130	0.255	0.26	0.61
**Histol. grade (G3 vs G1&G2)**	-0.034	0.257	0.018	0.90

* significant P-Values are given in bold
