# Supplementary material for: Homogeneous Datasets of Triple Negative Breast Cancers Enable the Identification of Novel Prognostic and Predictive Signatures
Source: PLoS One. 2011 Dec 29;6(12):e28403. doi: 10.1371/journal.pone.0028403 (PMC3248403; doi:10.1371/journal.pone.0028403)
Supplement: Table S5 — Pre-therapeutic samples from neoadjuvant treated TNBC. (PDF) [file pone.0028403.s014.pdf]

Supplementary Table S5: Pre-therapeutic samples from neoadjuvant treated TNBC

Dataset	Institution	Chemotherapy	TNBC	pCR (%)
MDA133	MDACC, Houston, TX	TFAC	36	18 (50.0)
Frankfurt-3	University of Frankfurt	TAC or TAC-NX	19	4 (21.1)
GSE18728	University of Washington/Seattle Cancer Care Alliance	Docetaxel/Capecitabine	8	3 (37.5)
GSE19697	Washington University School of Medicine St. Louis	E-Doc	24	5 (20.8)
GSE20194	MDACC, Houston, TX	paclitaxel, 5-fluorouracil, cyclo-phosphamide, doxorubicin	50	11 (22.0)
GSE20271	MDACC, Houston, TX	paclitaxel, 5-fluorouracil, cyclo-phosphamide, doxorubicin	54	11 (20.4)
Total			191	52 (27.2)
Validation:				
GSE16446	TOP-Trial; Institut Jules Bordet, Brussels	Epirubicin Mono	95	52 (27.2)
