# Supplementary material for: Homogeneous Datasets of Triple Negative Breast Cancers Enable the Identification of Novel Prognostic and Predictive Signatures
Source: PLoS One. 2011 Dec 29;6(12):e28403. doi: 10.1371/journal.pone.0028403 (PMC3248403; doi:10.1371/journal.pone.0028403)
Supplement: Table S6 — Clinical parameters of TNBC according to expression of the 264-probeset signature. (PDF) [file pone.0028403.s015.pdf]

Supplementary Table S6: Clinical parameters of TNBC according to expression of the 264-probeset signature

Parameter		Finding cohort (n=394) Total	Low expression*	High expression	P-Value	Validation cohort (n=261) Total	Low expression	High expression	P-Value#
Lymph node status	node negative	240	181	59		80	67	13	
	node positive	68	48	20	0.43	92	81	11	0.51
Age	> 50	158	114	44		123	93	30	
	≤ 50	151	117	34	0.30	93	70	23	1.00
Tumor size	≤ 2 cm	85	71	15		40	26	14	
	> 2 cm	224	160	63	0.058	184	152	32	**0.017**
Histological grading	G3	227	172	55		172	136	36	
	G1 & G2	82	63	19	0.88	60	48	12	1.00

* samples were stratified according to the highest quartile as in Kaplan-Meier and Cox regression analyses in Figure 2 and Table 2, respectively.

# significant P-Values are given in bold
