# Supplementary material for: Homogeneous Datasets of Triple Negative Breast Cancers Enable the Identification of Novel Prognostic and Predictive Signatures
Source: PLoS One. 2011 Dec 29;6(12):e28403. doi: 10.1371/journal.pone.0028403 (PMC3248403; doi:10.1371/journal.pone.0028403)
Supplement: Table S7 — Histological grade among samples in the finding and validation cohort. (PDF) [file pone.0028403.s016.pdf]

Supplementary Table S7: Distribution of histological grade in finding and validation cohort

Histological grade	finding cohort	validation cohort	total
G1	27 (8.7%)	25 (10.8%)	52 (9.6%)
G2	55 (17.8%)	35 (15.1%)	90 (16.6%)
G3	227 (73.5%)	172 (74.1%)	399 (73.8%)
Total	309 (85 missing)	232 (29 missing)	541 (114 missing)
