# Supplementary material for: Homogeneous Datasets of Triple Negative Breast Cancers Enable the Identification of Novel Prognostic and Predictive Signatures
Source: PLoS One. 2011 Dec 29;6(12):e28403. doi: 10.1371/journal.pone.0028403 (PMC3248403; doi:10.1371/journal.pone.0028403)
Supplement: Table S8 — Distribution of intrinsic molecular subtypes according to expression of the 264-probeset signature in TNBC. (PDF) [file pone.0028403.s017.pdf]

Supplementary Table S8: Distribution of intrinsic molecular subtypes according to expression of the 264-probeset signature in TNBC

	Basal-like	HER2-like	Luminal A	Normal breast -like	Unclassified	*Total*
SSP-prediction in cohort of all subtypes (n=1304 total, n=221 TNBC) *						
**264-probeset signature**	Centroid-Single Sample Predictor (data centered)					
highest quartile	53 (88.3%)	3 (5.0%)	0	4 (6.7%)	0	*60*
low expression	139 (86.3%)	2 (1.2%)	1 (0.6%)	19 (11.8%)	0	*161*
total	192(86.9%)	5 (2.3%)	1 (0.5%)	23 (10.4%)	0	*221*
SSP-prediction in pure TNBC cohort (n=579) †						
**264-probeset signature**	Centroid-Single Sample Predictor (data not centered)					
highest quartile	100 (64.9%)	2 (1.3%)	2 (1.3%)	0	50 (32.5%)	*154*
low expression	296 (69.6%)	6 (1.4%)	12 (2.8%)	4 (0.9%)	107 (25.5%)	*425*
total	396 (68.4%)	8 (1.4%)	14 (2.4%)	4 (0.7%)	157 (27.1%)	*579*

* A nearest centroid single sample predictor (SSP) method either with centering as described by Weigelt et al. (2010, Lancet Oncol; 11:339) was applied to seven larger datasets (Frankfurt, Mainz, NewYork, Stockholm, Transbig, Uppsala, Rotterdam) to assign a total of 1304 breast cancer samples to a molecular subtype (according to Hu et al. 2006, BMC Genomics, 7:96). The data for the 221 TNBC samples from this cohort that are included in our study are shown. The majority of the TNBC are assigned to the basal-like group by the centroid method (86.9%). In the group with high expression of the 264-probeset signature the proportion of "HER2-like" samples is somewhat higher (5.0 vs 1.2%) and those of the "Normal-breast-like" somewhat lower (6.7 vs 11.8%) but these differences did not reach statistical significance (P=0.24).

† Application of the centroid method to the cohort of 579 TNBC samples (without including non-TNBC samples). Data centering is not applicable to a complete ER negative cohort as previously described (Lusa et al. 2007, J Natl Cancer Inst. 99:1715). Therefore the uncentered version of the method described by Weigelt et al (2010, Lancet Oncol; 11:339) was applied for this cohort. This version of the method results in 27.1% of "unclassified" samples. Still the overall results are similar to those of the uncentered method applied to the dataset including non-TNBC samples above with most of the remaining samples assigned to "Basal-like" (68.4%). Similar to the results of the centered method above no significant difference of subtype assignment was observed between samples with high or low expression of the 264-probeset signature, respectively (P=0.27).

Detailed information and corresponding R-code for the applied SSP-methods can be downloaded from the authors of *Weigelt et al.* (2010, Lancet Oncol; 11:339) at:

http://rock.icr.ac.uk/collaborations/Mackay/centroid.correlations.Eset/ExpressionSet%20Nearest%20Centroid%20Correlations.pdf
