# Supplementary material for: Homogeneous Datasets of Triple Negative Breast Cancers Enable the Identification of Novel Prognostic and Predictive Signatures
Source: PLoS One. 2011 Dec 29;6(12):e28403. doi: 10.1371/journal.pone.0028403 (PMC3248403; doi:10.1371/journal.pone.0028403)
Supplement: Table S9 — 264 probeset supervised prognostic signature for TNBC from SAM. (PDF) [file pone.0028403.s018.pdf]

Supplementary Table S9: 264 probeset supervised prognostic signature for TNBC from SAM

**Affy_ID**	**GeneSymbol**	**SAM-Score**	**direction of prognostic value: poor / good**	**included in 264-probeset signature (FDR 25%)**	**included in 26-probeset signature (FDR <3.5%)**	
Poor prognosis markers						
211506_s_at	IL8	3.754	POOR	yes	**yes**	
211708_s_at	SCD	3.377	POOR	yes	**yes**	
39249_at	AQP3	3.308	POOR	yes	**yes**	
202859_x_at	IL8	3.299	POOR	yes	**yes**	
202627_s_at	SERPINE1	3.136	POOR	yes	**yes**	
212909_at	LYPDC1	3.118	POOR	yes	**yes**	
200737_at	PGK1	3.090	POOR	yes	**yes**	
204344_s_at	SEC23A	3.075	POOR	yes	**yes**	
205810_s_at	WASL	3.071	POOR	yes	**yes**	
217356_s_at	PGK1	3.031	POOR	yes	**yes**	
215779_s_at	HIST1H2BG	3.017	POOR	yes	**yes**	
212344_at	SULF1	3.008	POOR	yes	**yes**	
209875_s_at	SPP1	3.002	POOR	yes	**yes**	
219434_at	TREM1	2.982	POOR	yes	**yes**	
219508_at	GCNT3	2.966	POOR	yes	**yes**	
208881_x_at	IDI1	2.959	POOR	yes	**yes**	
215427_s_at	ZCCHC14	2.958	POOR	yes	**yes**	
214603_at	MAGEA2	2.956	POOR	yes	**yes**	
219875_s_at	PNAS-4	2.951	POOR	yes	**yes**	
204083_s_at	TPM2	2.948	POOR	yes	**yes**	
218468_s_at	GREM1	2.937	POOR	yes	**yes**	
204615_x_at	IDI1	2.902	POOR	yes	**yes**	
212354_at	SULF1	2.858	POOR	yes	**yes**	
218469_at	GREM1	2.836	POOR	yes	**yes**	
212353_at	SULF1	2.809	POOR	yes	**yes**	
202497_x_at	SLC2A3	2.797	POOR	yes	**yes**	
202539_s_at	HMGCR	2.745	POOR	yes	no	
214522_x_at	HIST1H3D	2.745	POOR	yes	no	
212942_s_at	KIAA1199	2.733	POOR	yes	no	
219773_at	NOX4	2.693	POOR	yes	no	
205680_at	MMP10	2.664	POOR	yes	no	
202912_at	ADM	2.656	POOR	yes	no	
201108_s_at	THBS1	2.654	POOR	yes	no	
210387_at	HIST1H2BG	2.653	POOR	yes	no	
205227_at	IL1RAP	2.648	POOR	yes	no	
201695_s_at	NP	2.645	POOR	yes	no	
217073_x_at	APOA1	2.595	POOR	yes	no	
201109_s_at	THBS1	2.595	POOR	yes	no	
215446_s_at	LOX	2.593	POOR	yes	no	
205443_at	SNAPC1	2.592	POOR	yes	no	
209978_s_at	LPA	2.591	POOR	yes	no	
201506_at	TGFBI	2.571	POOR	yes	no	
208577_at	HIST1H3C	2.569	POOR	yes	no	
219927_at	C14orf111	2.552	POOR	yes	no	
212210_at	DKFZP586J0619	2.539	POOR	yes	no	
202134_s_at	WWTR1	2.527	POOR	yes	no	
211162_x_at	SCD	2.526	POOR	yes	no	
218073_s_at	FLJ10407	2.524	POOR	yes	no	
212887_at	SEC23A	2.524	POOR	yes	no	
206354_at	SLCO1B3	2.522	POOR	yes	no	
201324_at	EMP1	2.520	POOR	yes	no	
212902_at	SEC24A	2.517	POOR	yes	no	

214978_s_at	PPFIA4	2.512	POOR	yes	no	
204338_s_at	RGS4	2.511	POOR	yes	no	
210089_s_at	LAMA4	2.502	POOR	yes	no	
214580_x_at	KRT6A	2.502	POOR	yes	no	
39248_at	AQP3	2.499	POOR	yes	no	
214725_at	RPESP	2.496	POOR	yes	no	
219104_at	RNF141	2.492	POOR	yes	no	
211840_s_at	PDE4D	2.489	POOR	yes	no	
200738_s_at	PGK1	2.482	POOR	yes	no	
203099_s_at	CDYL	2.480	POOR	yes	no	
207933_at	ZP2	2.477	POOR	yes	no	
202604_x_at	ADAM10	2.476	POOR	yes	no	
221291_at	ULBP2	2.472	POOR	yes	no	
214455_at	H2BFL	2.464	POOR	yes	no	
210431_at	ALPPL2	2.463	POOR	yes	no	
210845_s_at	PLAUR	2.454	POOR	yes	no	
211160_x_at	ACTN1	2.440	POOR	yes	no	
208547_at	HIST1H2BB	2.425	POOR	yes	no	
219837_s_at	CYTL1	2.414	POOR	yes	no	
201037_at	PFKP	2.409	POOR	yes	no	
200989_at	HIF1A	2.405	POOR	yes	no	
208490_x_at	HIST1H2BF	2.397	POOR	yes	no	
204298_s_at	LOX	2.387	POOR	yes	no	
201888_s_at	IL13RA1	2.387	POOR	yes	no	
220768_s_at	CSNK1G3	2.386	POOR	yes	no	
210405_x_at	TNFRSF10B	2.383	POOR	yes	no	
206315_at	CRLF1	2.382	POOR	yes	no	
217911_s_at	BAG3	2.378	POOR	yes	no	
219886_at	LRRIQ2	2.369	POOR	yes	no	
202628_s_at	SERPINE1	2.369	POOR	yes	no	
209305_s_at	GADD45B	2.366	POOR	yes	no	
210995_s_at	TRIM23	2.365	POOR	yes	no	
206409_at	TIAM1	2.362	POOR	yes	no	
201325_s_at	EMP1	2.359	POOR	yes	no	
219366_at	AVEN	2.357	POOR	yes	no	
210435_at	NBR2	2.355	POOR	yes	no	
205199_at	CA9	2.350	POOR	yes	no	
209803_s_at	PHLDA2	2.341	POOR	yes	no	
205499_at	SRPX2	2.334	POOR	yes	no	
214216_s_at	KIAA0217	2.330	POOR	yes	no	
217834_s_at	SYNCRIP	2.321	POOR	yes	no	
204883_s_at	HUS1	2.311	POOR	yes	no	
208527_x_at	HIST1H2BE	2.305	POOR	yes	no	
208129_x_at	RUNX1	2.303	POOR	yes	no	
214612_x_at	MAGEA6	2.302	POOR	yes	no	
215488_at	VMD2	2.299	POOR	yes	no	
206512_at	U2AF1L1	2.298	POOR	yes	no	
217428_s_at	COL10A1	2.292	POOR	yes	no	
216796_s_at	- - -	2.290	POOR	yes	no	
208427_s_at	ELAVL2	2.284	POOR	yes	no	
213506_at	F2RL1	2.283	POOR	yes	no	
208180_s_at	H4FH	2.282	POOR	yes	no	
215997_s_at	CUL4B	2.282	POOR	yes	no	
203000_at	STMN2	2.282	POOR	yes	no	
202388_at	RGS2	2.278	POOR	yes	no	
205523_at	HAPLN1	2.278	POOR	yes	no	
217675_at	LOC201501	2.273	POOR	yes	no	
203393_at	HES1	2.271	POOR	yes	no	
214702_at	FN1	2.268	POOR	yes	no	
209601_at	ACOX1	2.268	POOR	yes	no	
204475_at	MMP1	2.266	POOR	yes	no	
220623_s_at	TSGA10	2.258	POOR	yes	no	

216729_at	- - -	2.252	POOR	yes	no	
210990_s_at	LAMA4	2.251	POOR	yes	no	
210257_x_at	CUL4B	2.246	POOR	yes	no	
220759_at	FAM12B	2.242	POOR	yes	no	
212543_at	AIM1	2.241	POOR	yes	no	
219856_at	SARG	2.240	POOR	yes	no	
207696_at	FUT9	2.238	POOR	yes	no	
220031_at	ZA20D1	2.237	POOR	yes	no	
201110_s_at	THBS1	2.232	POOR	yes	no	
208394_x_at	ESM1	2.225	POOR	yes	no	
215649_s_at	MVK	2.218	POOR	yes	no	
206686_at	PDK1	2.206	POOR	yes	no	
206547_s_at	PPEF1	2.203	POOR	yes	no	
221679_s_at	ABHD6	2.200	POOR	yes	no	
204337_at	RGS4	2.196	POOR	yes	no	
210233_at	IL1RAP	2.191	POOR	yes	no	
201170_s_at	BHLHB2	2.185	POOR	yes	no	
211338_at	IFNA2	2.185	POOR	yes	no	
216650_at	LOC442165	2.184	POOR	yes	no	
201903_at	UQCRC1	2.179	POOR	yes	no	
202740_at	ACY1	2.179	POOR	yes	no	
208523_x_at	HIST1H2BI	2.175	POOR	yes	no	
208487_at	LMX1B	2.173	POOR	yes	no	
207370_at	IBSP	2.171	POOR	yes	no	
206113_s_at	RAB5A	2.171	POOR	yes	no	
215733_x_at	CTAG2	2.169	POOR	yes	no	
210805_x_at	RUNX1	2.168	POOR	yes	no	
205924_at	RAB3B	2.163	POOR	yes	no	
216672_s_at	MYT1L	2.153	POOR	yes	no	
206569_at	IL24	2.152	POOR	yes	no	
211617_at	ALDOAP2	2.151	POOR	yes	no	
222219_s_at	TLE6	2.150	POOR	yes	no	
207328_at	ALOX15	2.149	POOR	yes	no	
202363_at	SPOCK	2.147	POOR	yes	no	
202543_s_at	GMFB	2.147	POOR	yes	no	
213563_s_at	GCP2	2.145	POOR	yes	no	
216268_s_at	JAG1	2.142	POOR	yes	no	
210874_s_at	NAT6	2.139	POOR	yes	no	
209101_at	CTGF	2.139	POOR	yes	no	
211758_x_at	TXNDC9	2.139	POOR	yes	no	
201559_s_at	CLIC4	2.137	POOR	yes	no	
213640_s_at	LOX	2.133	POOR	yes	no	
206788_s_at	CBFB	2.133	POOR	yes	no	
219232_s_at	EGLN3	2.133	POOR	yes	no	
219328_at	DDX31	2.129	POOR	yes	no	
202540_s_at	HMGCR	2.128	POOR	yes	no	
209398_at	HIST1H1C	2.128	POOR	yes	no	
215574_at	- - -	2.124	POOR	yes	no	
218036_x_at	NMD3	2.121	POOR	yes	no	
210750_s_at	DLGAP1	2.118	POOR	yes	no	
40524_at	PTPN21	2.117	POOR	yes	no	
201275_at	FDPS	2.113	POOR	yes	no	
204614_at	SERPINB2	2.112	POOR	yes	no	
201043_s_at	ANP32A	2.109	POOR	yes	no	
201091_s_at	CBX3	2.108	POOR	yes	no	
37512_at	RODH	2.106	POOR	yes	no	
203108_at	GPCR5A	2.105	POOR	yes	no	
219948_x_at	FLJ21934	2.105	POOR	yes	no	
215646_s_at	CSPG2	2.098	POOR	yes	no	
213983_s_at	SCC-112	2.098	POOR	yes	no	
202057_at	KPNA1	2.098	POOR	yes	no	
208613_s_at	FLNB	2.097	POOR	yes	no	

220003_at	FLJ11004	2.095	POOR	yes	no	
201208_s_at	TNFAIP1	2.095	POOR	yes	no	
209822_s_at	VLDLR	2.095	POOR	yes	no	
209909_s_at	TGFB2	2.092	POOR	yes	no	
210467_x_at	MAGEA12	2.088	POOR	yes	no	
215432_at	BUCS1	2.085	POOR	yes	no	
209122_at	ADFP	2.084	POOR	yes	no	
210196_s_at	PSG1	2.083	POOR	yes	no	
210876_at	ANXA2	2.081	POOR	yes	no	
208546_x_at	HIST1H2BH	2.079	POOR	yes	no	
216414_at	- - -	2.079	POOR	yes	no	
212812_at	- - -	2.078	POOR	yes	no	
219733_s_at	SLC27A5	2.078	POOR	yes	no	
215254_at	DSCR1	2.074	POOR	yes	no	
207319_s_at	CDC2L5	2.070	POOR	yes	no	
211668_s_at	PLAU	2.068	POOR	yes	no	
201792_at	AEBP1	2.066	POOR	yes	no	
202238_s_at	NNMT	2.065	POOR	yes	no	
219356_s_at	SNF7DC2	2.060	POOR	yes	no	
210904_s_at	IL13RA1	2.059	POOR	yes	no	
221552_at	ABHD6	2.059	POOR	yes	no	
221009_s_at	ANGPTL4	2.058	POOR	yes	no	
214968_at	DDX51	2.055	POOR	yes	no	
201196_s_at	AMD1	2.055	POOR	yes	no	
212444_at	- - -	2.054	POOR	yes	no	
204845_s_at	ENPEP	2.053	POOR	yes	no	
208496_x_at	HIST1H3G	2.053	POOR	yes	no	
215464_s_at	TAX1BP3	2.052	POOR	yes	no	
212797_at	SORT1	2.051	POOR	yes	no	
204596_s_at	STC1	2.050	POOR	yes	no	
210623_at	LOC51035	2.050	POOR	yes	no	
216915_s_at	PTPN12	2.050	POOR	yes	no	
208144_s_at	PP1345	2.049	POOR	yes	no	
214469_at	HIST1H2AE	2.049	POOR	yes	no	
205479_s_at	PLAU	2.044	POOR	yes	no	
217448_s_at	C14orf92	2.044	POOR	yes	no	
216607_s_at	CYP51A1	2.043	POOR	yes	no	
210619_s_at	HYAL1	2.042	POOR	yes	no	
214540_at	HIST1H2BO	2.039	POOR	yes	no	
216549_s_at	TBC1D22B	2.039	POOR	yes	no	
219478_at	WFDC1	2.037	POOR	yes	no	
209624_s_at	MCCC2	2.034	POOR	yes	no	
212575_at	C19orf6	2.034	POOR	yes	no	
222379_at	KCNE4	2.031	POOR	yes	no	
211924_s_at	PLAUR	2.030	POOR	yes	no	
221933_at	NLGN4X	2.030	POOR	yes	no	
220414_at	CALML5	2.029	POOR	yes	no	
215976_at	DBC1	2.026	POOR	yes	no	
210546_x_at	CTAG1B	2.025	POOR	yes	no	
216712_at	SLC25A30	2.020	POOR	yes	no	
220106_at	NPC1L1	2.020	POOR	yes	no	
212898_at	KIAA0406	2.019	POOR	yes	no	
202213_s_at	CUL4B	2.019	POOR	yes	no	
216830_at	- - -	2.017	POOR	yes	no	
204325_s_at	NF1	2.017	POOR	yes	no	
215423_at	- - -	2.016	POOR	yes	no	
205228_at	RBMS2	2.013	POOR	yes	no	
208083_s_at	ITGB6	2.011	POOR	yes	no	
218182_s_at	CLDN1	2.011	POOR	yes	no	
Good prognosis markers						
209591_s_at	BMP7	-3.405	GOOD	yes	no	
221671_x_at	IGKC	-3.118	GOOD	yes	no	

211333_s_at	FASLG	-3.107	GOOD	yes	no	
221651_x_at	IGKC	-3.097	GOOD	yes	no	
211259_s_at	BMP7	-3.041	GOOD	yes	no	
218872_at	TSC	-3.019	GOOD	yes	no	
209460_at	ABAT	-3.010	GOOD	yes	no	
217455_s_at	SSTR2	-2.890	GOOD	yes	no	
208479_at	KCNA1	-2.883	GOOD	yes	no	
205890_s_at	UBD	-2.860	GOOD	yes	no	
221087_s_at	APOL3	-2.843	GOOD	yes	no	
210321_at	GZMH	-2.820	GOOD	yes	no	
214510_at	GPR20	-2.777	GOOD	yes	no	
211430_s_at	IGH@	-2.761	GOOD	yes	no	
214567_s_at	XCL1	-2.759	GOOD	yes	no	
217143_s_at	TRA@	-2.722	GOOD	yes	no	
207796_x_at	KLRD1	-2.690	GOOD	yes	no	
214677_x_at	IGL@	-2.688	GOOD	yes	no	
203915_at	CXCL9	-2.681	GOOD	yes	no	
208498_s_at	AMY2A	-2.679	GOOD	yes	no	
219605_at	ZNF3	-2.677	GOOD	yes	no	
209138_x_at	IGLC2	-2.661	GOOD	yes	no	
209590_at	BMP7	-2.654	GOOD	yes	no	
204781_s_at	FAS	-2.653	GOOD	yes	no	
217378_x_at	LOC391427	-2.641	GOOD	yes	no	
218062_x_at	CDC42EP4	-2.631	GOOD	yes	no	
212272_at	LPIN1	-2.624	GOOD	yes	no	
203608_at	ALDH5A1	-2.621	GOOD	yes	no	
207747_s_at	DOK4	-2.619	GOOD	yes	no	
